# The State of the Art of Telemedicine Implementation Architecture: Rapid Umbrella Review of Systematic Reviews

**DOI:** 10.2196/70276

**Published:** 2025-06-09

**Authors:** Che Katz, José María Ruiz, Francesc Saigí-Rubió, David Novillo-Ortiz

**Affiliations:** 1 Universitat Oberta de Catalunya Faculty of Health Sciences Barcelona Spain; 2 World Health Organization Regional Office for Europe Division of Country Health Policies and Systems Copenhagen Denmark

**Keywords:** telemedicine, implementation research, umbrella review, knowledge translation, tools, delivery of health care

## Abstract

**Background:**

The global push to scale up telemedicine services is challenged by complex, multilevel, multifaceted implementation and a lack of consensus on what the evidence-based essential building blocks of implementation are.

**Objective:**

We aimed to evaluate the evidence base supporting telemedicine implementation knowledge tools; identify shared conceptual constructs and outliers; and formulate recommendations to guide the design, development, and optimization of telemedicine services.

**Methods:**

We conducted implementation research using a rapid umbrella review, that is, an overview of systematic reviews, in accordance with PRISMA (Preferred Reporting Items for Systematic Reviews and Meta-Analyses). In total, we searched 3 databases (PubMed, Web of Science, and Scopus) for studies focusing on telemedicine implementation frameworks, models, and tools, collectively referred to as “knowledge tools.” Reviews meeting the operational definition of a systematically undertaken, secondary evidence synthesis, such as systematic and scoping reviews, and those published from January 2018 to May 2024 were considered. A meta-aggregative qualitative analysis was undertaken, comprising inductive thematic synthesis.

**Results:**

In total, 18 reviews were selected, encompassing 973 primary studies. Global perspectives were reflected in 61% (n=11) of the reviews, while 33% (n=6) focused on low- and middle-income country contexts. The primary research included in the reviews represented 63 countries, spanning the Americas, Europe, Africa, the Middle East, and Asia and the Pacific. Findings indicated substantial heterogeneity across the identified telemedicine implementation theories, models, and frameworks. However, following evidence synthesis, considerable convergence was observed, highlighting a state-of-the-art understanding of the essential requirements for a national telemedicine implementation ecosystem. These were categorized into “process” and “thematic” dimensions. Process dimensions included readiness and needs assessment, road map and planning, managing change, implementing telemedicine services, and continuous improvement and measuring performance. Thematic dimensions covered human and sociocultural aspects; organization, operations, management, and leadership; communication and coordination; policy, legal, and financial considerations; clinical health condition and quality of care; and the wider context.

**Conclusions:**

The findings of this study inform a pressing translational research knowledge gap in telemedicine implementation, hindering the implementation of high-quality, sustainable, and scalable telemedicine systems. The study contributes to building global consensus on the state of the art of key constructs in telemedicine implementation and recommends that future research focus on field-testing the evidence-based implementation tools to evaluate their usability and adaptability across diverse telemedicine contexts.

## Introduction

### Background

The unprecedented growth of telemedicine services during the COVID-19 pandemic is acknowledged to have played a vital role in saving the global health system from collapse [1,2]. The impetus created by this crisis provided a compelling evidence base for the value added that telemedicine services contribute in terms of strengthening health service delivery. There is now considerable evidence of the promise that high-quality telemedicine services hold for supporting the multitude of challenges faced by health services around the world, contributing to improved health outcomes, quality, and health care provider and patient satisfaction [3-5], and to reducing costs [6], improving health service access for rural and remote communities [7,8], and protecting the environment [9]. However, the challenges faced by countries around the world to consolidate, sustain, and expand national telemedicine services [3,10,11] point to the multilevel and multifaceted complexity of this innovation.

Telemedicine is recognized as a fundamental pillar in the digital transformation of health systems, with a strong global push for scale-up [12-15]. However, defining “telemedicine” is becoming increasingly abstruse, with no universally agreed definition [16,17], and many related terms, such as “telehealth,” are used interchangeably by some and distinctly by others [18]. Moreover, the boundaries between telemedicine and other digital health interventions are becoming increasingly blurred, with telemedicine progressively being implemented alongside other “digital health interventions,” such as wearable devices and artificial intelligence. This study adopts the World Health Organization (WHO) definition of telemedicine, subsumed under “digital health,” encompassing remote clinical synchronous or asynchronous communication between those separated by distance, either client-to–service provider or service provider–to–service provider [19]. This definition includes the internet-based transfer of medical data, such as images, patient records, and clinical information, from remote monitoring devices. In this study, telemedicine is examined as an integrated part of the health ecosystem rather than as a stand-alone, siloed intervention.

There is now considerable evidence of the barriers to, facilitators of, and lessons learnt from the implementation of telemedicine across the world [3,20,21]. There is also an increasing number of knowledge tools (KTs) [19,22-28], defined as resources that help translate evidence into practice [29], often found in the form of conceptual frameworks, models, and theories [30]. However, there is considerable heterogeneity across these KTs, as well as a lack of consensus about the building blocks, taxonomy, and definitional boundaries of the constructs of telemedicine implementation along the life cycle [31,32]. This challenges telemedicine stakeholders, who need user-friendly knowledge mobilization resources to support them in their urgent task of designing, developing, and optimizing telemedicine services [32-34].

### Objectives

This implementation research, rapid umbrella review, and translational study contributes to an understanding of the requirements for the development of telemedicine systems. In this study, “implementation research” as opposed to “implementation science” is understood to focus on approaches for promoting the integration of evidence into the design and development of telemedicine interventions [35]. The study aimed to identify the evidence base for the constructs of telemedicine service implementation at scale by conducting an overview of systematic reviews of telemedicine implementation theories, models, and frameworks to improve telemedicine service development and outcomes, answering the research question: *What is the state of the art of the implementation constructs of telemedicine architecture?* Furthermore, this study aimed to assess the evidence base supporting telemedicine implementation KTs; identify shared conceptual constructs and outliers; and formulate recommendations to guide telemedicine services’ design, development, and optimization.

## Methods

### Study Design

A rapid umbrella review was undertaken to consolidate knowledge on the building blocks or constructs of telemedicine implementation. Its adoption was prompted by urgent requests for technical guidance received by the WHO Collaborating Centre for eHealth at the Universitat Oberta de Catalunya, in response to needs expressed by national telemedicine leaders in lower-resourced countries. These focused on strategic advice regarding the core constructs of a telemedicine ecosystem to support national scale-up efforts. In addressing these demands, a lack of expert consensus and significant knowledge gaps were identified [3,10,11,31,32]. The combination of urgency, limited resources, and the need for timely, actionable outputs justified the selection of a rapid umbrella review as the most appropriate methodological approach [5,12-15]. On the other hand, the umbrella review approach was well suited to the substantial body of secondary evidence from systematic reviews focused on telemedicine implementation constructs. Umbrella reviews have grown in popularity in public health [36,37], and by modifying, streamlining, and accelerating the research process, the “rapid” umbrella review approach provides a robust, resource-efficient advantage [38,39], notwithstanding some limitations [40]. This accelerated research approach has responded to ongoing national telemedicine implementation efforts by promptly integrating state-of-the-art evidence—understood as the synthesis of current knowledge [41,42]—while also contributing to future telemedicine knowledge translation.

Our study was primarily guided by the WHO *Rapid Review to Strengthen Health Policy and Systems* [38] and was further informed by additional methodological guidance relevant to rapid and umbrella reviews, complex interventions, qualitative and mixed methods analysis, and implementation science [36,37,40,43,44]. The study followed the reporting requirements of the PRISMA (Preferred Reporting Items for Systematic Reviews and Meta-Analyses) guidelines (2020) [45] along with the extension for complex interventions [46]. The study was registered in PROSPERO on February 13, 2024 (2024 CRD42024512516; Multimedia Appendix 1).

### Search Strategy

Search term keywords were defined, informed by existing evidence synthesis, Medical Subject Headings (MeSH), and the technical support of a specialist librarian. On May 20, 2024, a multifield search was adapted to the indexing language of 3 databases (PubMed, Web of Science, and Scopus; Multimedia Appendix 2). The database search was supplemented by recommendations from telemedicine experts and a search of systematic review repositories (including Joanna Briggs Institute Evidence Synthesis, Cochrane Database of Systematic Reviews, PROSPERO, Database of Abstracts of Reviews of Effects, Epistemonikos, and Campbell Collaboration). The primary researcher (CK) performed the search, and all the articles were imported into the Covidence (Veritas Health Innovation) software and deduplicated.

### Selection Criteria

Screening was undertaken in a 2-stage process from May 20, 2024, to June 1, 2024, based on predefined inclusion criteria (Textbox 1). After pilot-testing the criteria for mutual understanding, 2 researchers (CK and JMR) independently screened titles and abstracts against the criteria, and conflicts were resolved by the third researcher (FSR). Shortlisted reviews were then assessed based on their full text by the same review team at the second stage of selection. During the selection process, several consensus meetings were held to ensure rigor, quality, and consistency in that process.

To integrate the full spectrum of evidence relevant to the research objectives, the search terms struck a balance between specificity and broader inclusivity, as recommended by the umbrella review methodological guidance [36]. The “study design” criteria included research methodologies that demonstrated systematic and robust secondary evidence synthesis, specifically “systematic reviews” and “scoping reviews” (referred to as SRs), with all other study designs excluded. The search on “telemedicine” included related terms, such as “telecare” and “telehealth,” as well as broader terms like “remote consultation,” “eHealth,” and “digital health.” These broader terms were further screened and only considered for inclusion when primary research on telemedicine was present. Studies that explored telemedicine implementation constructs at scale, reaching multiple populations (irrespective of setting, specialty, group of patients, condition, or disease), were included, while studies reaching a single population were excluded. Given the increasing integration of telemedicine into the wider digital health ecosystem, digital health studies that explored implementation constructs and incorporated telemedicine were included, whereas those with no mention of telemedicine were excluded. Studies before and during implementation were included, but those focusing on postimplementation outcomes were excluded as these were being synthesized in a concurrent meta-analysis undertaken by the same organization. Finally, to meet the state-of-the-art research objectives, the search was limited to studies published between January 1, 2018, and May 20, 2024. No additional filters were applied. Three main factors informed the selection of this timeframe. First, this period saw an unprecedented surge in evidence generation and global policy directives promoting the development and scale up of national telemedicine initiatives [5,19,47-49]. Second, there was a marked acceleration in technological advancements, including the widespread availability of high-speed internet, the ubiquity of smartphones, the expansion of remote monitoring devices, and the emergence of artificial intelligence. These innovations have significantly enhanced the scalability of telemedicine while also introducing new implementation challenges and opportunities [13]. Third, the volume of telemedicine research increased substantially during this period, reflecting growing scientific interest and activity in the field [50-53].

**Inclusion and exclusion criteria.**

**Inclusion criteria**

- Population
  Telemedicine interventions that reach multiple populations irrespective of settings, specialty, group of patients, or multiple conditions or diseases
- Intervention
  Explores telemedicine implementation at scale (ie, national or regional level)
  Explores constructs or architecture of telemedicine implementation (ie, design, development, maturity models, readiness, needs assessments, guidelines, models, frameworks, and other knowledge tools)
- Comparator
  Traditional in-person services and consultations or no comparator
- Outcome
  Explores preimplementation or during implementation constructs of telemedicine and synthesizes telemedicine implementation constructs into a form of a knowledge tool (ie, guidelines, models, and frameworks)
- Study design
  Systematic reviews or scoping reviews of qualitative, quantitative, or mixed methods (including, realist, integrative reviews, evidence maps, rapid reviews, narrative reviews, concept analyses reviews, or umbrella reviews), demonstrating an explicit and reproducible methodology (ie, a statement of objective, predefined inclusion criteria, systematized search strategy of at least 2 databases, and peer-reviewed)
- Characteristics
  Date restriction: January 1, 2018, to May 20, 2024
  No language restrictions imposed
  Full text available

**Exclusion criteria**

- Population
  Telemedicine interventions that reach a single population (ie, focus on a single demographic profile, a single condition, or a disease)
- Intervention
  Not related to telemedicine implementation
  Does not explore constructs or architecture of telemedicine implementation
  Focuses on a specific telemedicine intervention or disease (ie, imaging, weight loss, or diabetes)
  Focuses on telemedicine interventions that are localized or not reaching multiple levels (ie, in a single or small group of health facilities)
  Primarily focuses on telemedicine outcomes (ie, clinical outcomes, health system outcomes, or evaluation of telemedicine services)
  Constructs of implementation of other types of digital health interventions with no inclusion of telemedicine (ie, digital health, eHealth, mobile health, ubiquitous health, artificial intelligence, and robotics)
- Comparator
  Not applicable
- Outcome
  Focuses on telemedicine postimplementation measures, outcomes, or performance indicators
  No evidence of synthesis of implementation constructs into a form of a knowledge tool
- Study design
  Reviews not meeting the operational definition of systematic reviews or scoping reviews
  Study protocols
  Any other types of articles (ie, editorials and primary research).
- Characteristics
  Date restriction: any articles before January 1, 2018
  Full text not available
  No duplicates

### Critical Appraisal

Critical appraisal, although not mandatory for rapid reviews [38], was undertaken as a good practice measure. On completion of the second screening, the final sourced SRs were appraised in duplicate by the same review team as the screening, using the Joanna Briggs Institute *Critical Appraisal Checklist for Systematic Reviews and Research Synthesis* [36]. These were scored on a 4-point scale of “very good,” “good,” “fair,” and “poor.” Those scoring “poor” were rejected due to inadequate methodological quality.

### Data Extraction and Management

Data extraction was performed by the principal researcher (CK), with key variables extracted in duplicate by JMR, using a predefined template in Excel (Microsoft Corporation). A third researcher (FSR) verified the data. Multimedia Appendix 3 provides the extraction table template, which includes the following: citation, geographic characteristics, methodology, objectives, search strategy, review findings, critical appraisal score, and a summary of the characteristics of the primary research of each SR (including the number of studies, years of publication, methodologies, country, settings, type of telemedicine interventions, clinical specialties, and theoretical underpinnings). Where possible, data were extracted verbatim to remain faithful to the authors’ original intent.

### Data Synthesis

The analysis was primarily qualitative, combining a meta-aggregative approach with narrative, thematic, and framework synthesis [36,37] and summarizing and explaining the findings in tables and text. Analysis followed the principles of reflexivity, acknowledging the inherent subjectivity of the researcher, through a 6-step iterative process of familiarization, coding, thematic categorization, theme review, further definition, and reporting [54,55]. This included exploring similarities, variations, and exceptions across reviews. The review findings were coded in ATLAS.ti (version 25; Scientific Software Development) by the principal reviewer (CK) using thematic inductive analysis. The research team had regular discussions over the course of the analysis to scrutinize assumptions in the interpretation of the data. Consideration was also given to the impact of primary study duplication across reviews. Due to the broad nature of the research question and the heterogeneity of the reviews, a meta-analysis was not possible, nor relevant, although characteristics of the studies were summarized in descriptive statistics and frequency tables. The findings of the SR authors were synthesized, without attempting to reinterpret findings, drawing out generalizable conclusions to guide practice and policy [36].

In the analysis, attention was paid to the methodological considerations of conducting evidence synthesis of complex adaptive health systems [37] comprising multilevel components and “fuzzy” boundaries [56], with a diverse range of stakeholders that interact in nonlinear dynamic ways. Such systems are known to be unpredictable in the way they self-organize and respond to change. Indeed, understanding “context” and how to integrate it into the analysis was particularly important [57].

### Modifications

Our study fully adhered to the allowable methodological adaptations outlined in rapid review guidance, ensuring both methodological rigor and the timely delivery of findings [38,43]. As required, all modifications have been transparently reported in this section. First, the search was scoped, applying a year of publication filter (2018 to 2024) to ensure state-of-the-art evidence in recognition of the exponential growth of telemedicine research since the COVID-19 pandemic [51], and the search strategy was limited to 3 databases, with a supplementary search of review repositories. Second, coding was undertaken by one researcher, acknowledging the inherent interpretive and subjective nature of thematic analysis [55], and the research team enriched the analysis through iterative collaborative reflection. Third, the research protocol objectives were further strengthened to more explicitly consider “knowledge translation” tools and “context.” Alternations were documented in a “decision log.” All deviations from standard umbrella review procedures were consistent with accepted rapid review methodologies and enabled the generation of timely, high-quality findings to address an urgent knowledge gap. Nonetheless, as is inherent in the rapid review approach, the interpretation of findings should be approached with appropriate caution.

## Results

### Search Results

**Figure 1:**
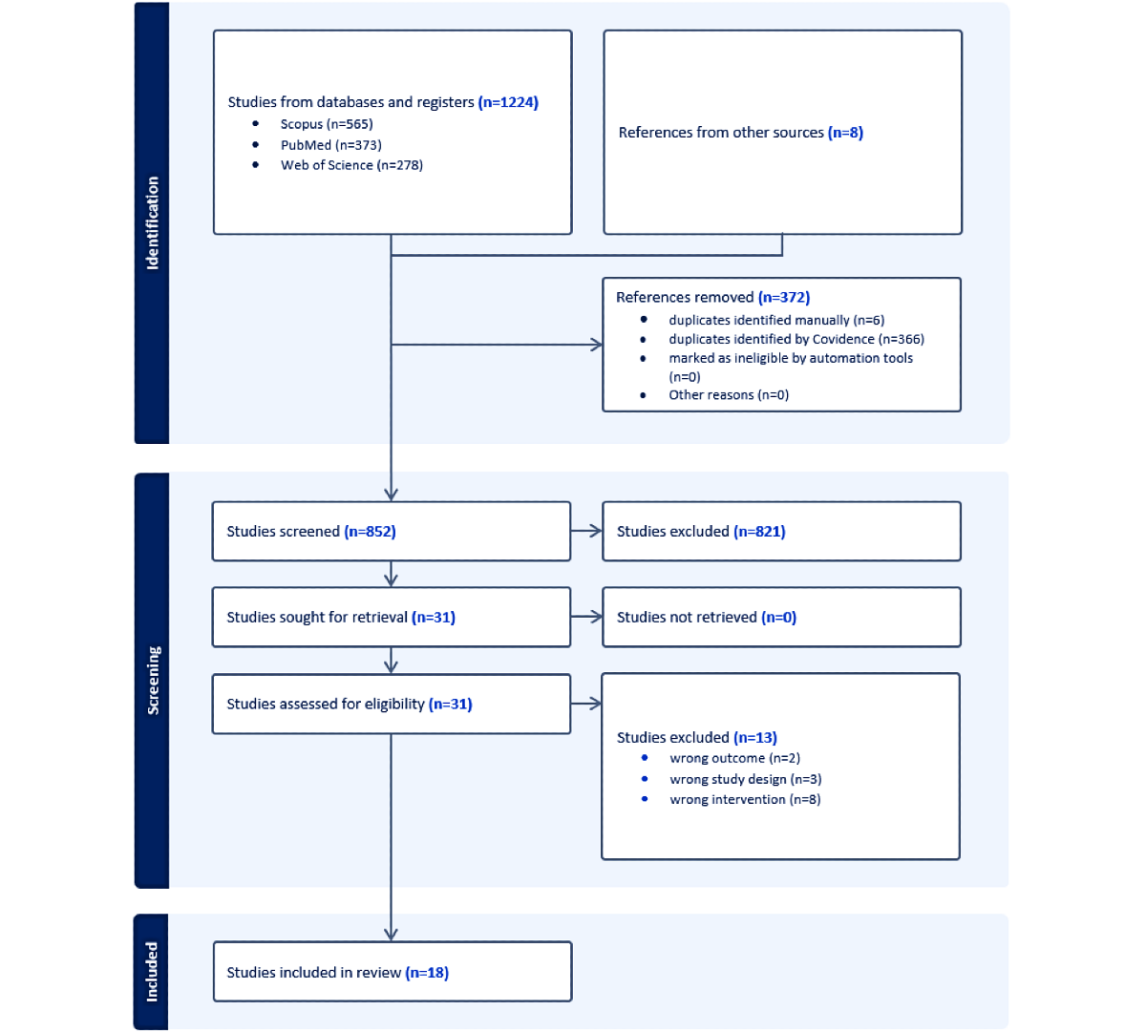
PRISMA diagram.

From the database search, we identified 1224 articles, 565 (46.16%) from Scopus, 373 (30.47%) from PubMed, 278 (22.71%) from Web of Science, and 8 from other supplementary searches. Of these, 372 (30.39%) duplicates were removed, leaving 852 (69.61%) articles. After being screened by title and abstract, 821 (96.4%) of those studies were excluded. The remaining 31 (3.6%) articles were screened for full text, and a further 13 (42%) studies were excluded: 8 (61%) for reasons of wrong intervention, 3 (24%) for wrong study design, and 2 (15%) for wrong outcomes (Figure 1). Finally, 18 (58%) SRs were selected and critically appraised, all meeting the minimum quality standard for inclusion. On the 4-point scale, 15 (83%) of the included reviews were assessed as good and 3 (17%) as fair, with none of the reviews meeting the highest or lowest quality standard (Multimedia Appendix 4 [58-75]).

### Characteristics

#### Overview of Reviews

The 18 SRs sourced are summarized in Table 1.

Telemedicine was the focus of 11 (61%) of the included reviews, while 7 (39%) took a broader digital health perspective, all including telemedicine as a central element. The methodological approach to evidence synthesis was evenly distributed, comprising 8 (44%) systematic reviews, 8 (44%) scoping reviews, and 2 (11%) less robust systematic literature reviews. The reviews were published in a variety of health-related journals: 3 (17%) in the *Journal of Medical Internet Research*, 2 (11%) in *BMC Health Services Research*, and the rest (13/18, 72%) distributed across a variety of other public health, global health, telemedicine, and digital health–focused academic journals. Analyzing the quality of the journals where the SRs were published, by year of publication, 12 (6%) were published in high-ranking first quartile journals, 4 (22%) in second quartile journals, and 2 (11%) in third quartile publications.

**Table 1:** Characteristics of included reviews.

References	Country focus of the review	Purpose of the review	Thematic focus	Primary studies, n	Results as stated in the abstract
[58]	Global	To analyze what the relevant stakeholders consider enablers and impediments of trust in digital health	Digital health	278	A total of 278 qualitative, quantitative, mixed methods, and intervention studies, published between 1998 and 2017 from 40 countries, were evaluated. Patients and health care professionals were the 2 most prominent stakeholders of “trust” in digital health; a third—health administrators—was substantially less prominent. The analysis identified cross-cutting personal, institutional, and technological elements of trust that broadly cluster into 16 enablers (altruism, fair data access, ease of use, self-efficacy, sociodemographic factors, recommendation by other users, usefulness, customizable design features, interoperability, privacy, initial face-to-face contact, guidelines for standardized use, stakeholder engagement, improved communication, decreased workloads, and service provider reputation) and 10 impediments (excessive costs, limited accessibility, sociodemographic factors, fear of data exploitation, insufficient training, defective technology, poor information quality, inadequate publicity, time-consuming, and service provider reputation) to trust in digital health.
[59]	Global	To summarize the opportunities and challenges of using telehealth in health care delivery during the COVID-19 pandemic	Telemedicine	112	A total of 112 unique opportunities of telehealth application during the pandemic were categorized into four key themes—(1) clinical, (2) organizational, (3) technical, and (4) social—which were further divided into 11 initial themes and 26 unique concepts. Furthermore, 106 unique challenges were categorized into six key themes—(1) legal, (2) clinical, (3) organizational, (4) technical, (5) socioeconomic, and (6) data quality—which were divided into 16 initial themes and 37 unique concepts. Clinical opportunities and legal challenges were the most frequent opportunities and challenges, respectively.
[60]	Middle East	To review the progress of the use and adoption of telemedicine in Middle Eastern countries and to identify the key dimensions affecting the progress of telemedicine in these countries	Telemedicine	43	The results showed that progress made in the use of telemedicine was insufficient and varied across Middle Eastern countries. Certain cultural, financial, organizational, individual, technological, legal, and regulatory challenges were found to prevent telemedicine from being fully used to the point where the full range of medical services could be provided. For example, physician and patient resistance, poor infrastructure, lack of funding, poor system quality, and lack of information technology training were associated with the low adoption of telemedicine in the region.
[61]	Global	To investigate models and videoconference software platforms for providing virtual care and barriers to and facilitators of accepting and using virtual care	Telemedicine	20	A total of 20 studies were included in the review. The findings extracted from the articles showed 4 main topics: “virtual care delivery models,” “videoconference software platforms to provide virtual care,” “virtual care delivery challenges,” and “virtual care implementation facilitators.” Thus, with the development of emerging digital technologies, unique opportunities to provide virtual care and improve the provision of health services have been created in the health care system worldwide.
[62]	Global	To systematically determine the challenges of and barriers to using health information technology in home care and to identify possible solutions	Telemedicine	47	A total of 47 studies were included. The main barriers and challenges based on the SURE^a^ framework were categorized into facilities (n=35); legislation or regulations (n=19); knowledge and skills (n=18); attitudes regarding program acceptability, appropriateness, and credibility (n=16); financial resources (n=10); motivation to change (n=9); and external communication (n=8). Studies mostly provided solutions regarding challenges related to the usability and functionality of applied technology.
[63]	Low- and middle-income countries	To identify and describe the evidence available in open-source data and existing literature regarding the implementation of digital health applications in 5 exemplar lower-middle-income countries (Bangladesh, India, Indonesia, Malaysia, and Pakistan)	Digital health	118	A total of 118 studies (2015-2021) and 114 supplementary web-based news articles and national statistics were included. Digital health policy was available in all countries, but scarce skilled labor, lack of legislation or interoperability support, and interrupted electricity and internet services were limitations. Older patients, women, and those living in rural areas were least likely to have access to ICT^b^ infrastructure. Renewable energy has potential in enabling digital health care. Low use mobile data and voice service packages are relatively affordable options for mHealth^c^ in the 5 countries.
[64]	Global	To explore which mobile health frameworks are used the most and to understand clinicians’ adoption of mHealth as well as to identify potential shortcomings in existing frameworks	Digital health	50	The most commonly used frameworks were different forms of extensions of the TAM^d^ (17/50, 34%), the DOI^e^ Theory (8/50, 16%), and different forms of extensions of the Unified Theory of Acceptance and Use of Technology (6/50, 12%). Some studies used a combination of the TAM and DOI frameworks (3/50, 6%), whereas others used the Consolidated Framework for Implementation Research (3/50, 6%) and the Sociotechnical Systems Theory (2/50, 4%). The factors cited in more than 20% of the studies were usefulness, output quality, ease of use, technical support, data privacy, self-efficacy, attitude, organizational inner setting, training, leadership engagement, workload, and workflow fit. Most factors could be linked to one framework or another, but there was no single framework that could adequately cover all relevant and specific factors without some expansion.
[65]	Global	To review and synthesize reported opportunities, challenges, and lessons learned in the scale-up, spread, and sustainability of video consultations and to identify transferable insights that can inform policy and practice	Telemedicine	13	A total of 13 articles describing 10 different video consultation services in 6 regions were included, covering the following: (1) video-to-home services, connecting service providers directly to the patient, (2) hub-and-spoke models, connecting a service provider at a central hub to a patient at a rural center, and (3) large-scale top-down evaluations scaled up or spread across a national health administration. Services covered rehabilitation, geriatrics, cancer surgery, diabetes, and mental health, as well as general specialist care and primary care. Potential enablers of spread and scale-up included embedded leadership and the presence of a telehealth champion, appropriate reimbursement mechanisms, user-friendly technology, preexisting staff relationships, and adaptation (of technology and services) over time. Challenges tended to be related to service development, such as the absence of a long-term strategic plan, resistance to change, cost and reimbursement issues, and technical experience of staff. There was limited articulation of the challenges relating to the scale-up and spread of video consultations. This was combined with a lack of theorization, with articles tending to view spread and scale-up as the sum of multiple technical implementations rather than theorizing the distinct processes required to achieve widespread adoption.
[66]	Global	To examine what and how change management practices have been applied to telemedicine service implementation, spanning a variety of health care areas and countries	Telemedicine	48	From 48 articles, 16 change management practices were identified relating to either strategic or operational aspects of telemedicine implementation. The key change management practices recognized in the broader change management literature as being essential to successful and sustained change were not commonly reported in telemedicine implementation studies. Drawing on change management literature, the study provides a comprehensive process-based, research-informed, organizing framework to guide future telemedicine service implementation and research.
[67]	Global	To propose a framework for the design and evaluation of digital health interventions that describes a typical life cycle of a digital health intervention and recommends relevant evaluation criteria and implementation barriers to be considered for each phase of the life cycle	Digital health	36	The study identified four life cycle phases of digital health interventions: (1) preparation, (2) optimization, (3) evaluation, and (4) implementation. It also identified 331 evaluation criteria and 98 implementation barriers. These were aligned into the DEDHI^f^ framework (which is based on the MOST^g^ model). Some barriers were found not to be present in the DEDHI framework, such as missing benefits, cooperation, and responsibilities, as well as the characteristics of the disease involved, which would hinder the use of digital health interventions in general.
[68]	United States	To determine the underlying themes regarding facilitators and barriers experienced by ambulatory care organizations when implementing telehealth during the COVID-19 pandemic	Telemedicine	24	Researchers identified 3 barriers impacting the implementation and use of telehealth resources: patient telehealth limitations; lack of clinical care telehealth guidelines; and training, technology, and financial considerations.
[69]	Low- and middle-income countries	To critically analyze published eHealth readiness assessment frameworks and to establish if any of them are appropriate for broad application in low- and middle-income countries	Digital health	13	A total of 8 types of eHealth readiness were identified, and no article directly addressed all of these. The frameworks were based upon varying assumptions and perspectives. There was no underlying unifying theory underpinning the frameworks. Few articles assessed government and societal readiness or cultural readiness; all were important in the low- and middle-income countries. While the shortcomings of existing frameworks were highlighted, most articles contained aspects that were relevant and could be drawn on when developing a framework and assessment tools for the low- and middle-income countries. What emerged was the need to develop different assessment tools for the various stakeholder sectors. This is an area that needs further research before attempting to develop a more generic framework for the low- and middle-income countries.
[70]	Low- and middle-income countries	To explore existing eHealth policy frameworks and propose a framework suitable for the development of eHealth policy in the context of low- and lower-middle-income countries	Digital health	11	Most of these publications did not develop or synthesize new frameworks for eHealth policy implementation, but rather introduced eHealth implementation frameworks, explained policy dimensions, identified and extracted relevant components of existing frameworks, or pointed out legal or other relevant eHealth implementation issues.
[71]	Global	To examine the structural elements that are relevant for implementing remote patient monitoring integrated care	Telemedicine	28	A total of 28 articles were included, covering 9 conceptual models and 19 real-life initiatives. In total, 18 structural elements of remote patient monitoring integrated care implementation were identified among conceptual models, defining a structure for assessing real-life initiatives. Of those initiatives, 78.9% referred to at least 10 structural elements, with patient education and self-monitoring promotion, multidisciplinary workforce, ICT and telemonitoring devices, and health indicators measurement being present in all studies, and therefore being core elements to the design of remote patient monitoring initiatives.
[72]	Global	To identify the methodological frameworks used worldwide for digital health technology assessment, to determine what domains are being considered, and to generate, through a thematic analysis, a proposal for a methodological framework based on the most frequently described domains in the literature	Digital health	26	Of the 26 studies included, 102 methodological frameworks designed for dHTA^h^ were identified. These frameworks revealed great heterogeneity due to their different structures, approaches, and items to be considered in dHTA. In addition, different wording used to refer to similar concepts was identified. Through thematic analysis, this heterogeneity was reduced. In the first phase of the analysis, 176 provisional codes related to the different assessment items emerged. In the second phase, these codes were clustered into 86 descriptive themes, which, in turn, were grouped in the third phase into 61 analytic themes and organized through a vertical hierarchy of three levels: level 1 formed by 13 domains, level 2 formed by 38 dimensions, and level 3 formed by 11 subdimensions. From these 61 analytic themes, a proposal for a methodological framework for dHTA was developed.
[73]	Global	To summarize the current state of facilitating and inhibiting factors that may influence the uptake of telerehabilitation	Telemedicine	28	A total of 28 studies (timespan from 2012 to 2023) were included. The most relevant barriers identified were technical issues and a lack of technical skills. The factors considered most favorable to implementation were patients’ motivation and the involvement of high-level leaders. The results provided clear indications of factors that inhibited and facilitated implementation and showed that further research was needed.
[74]	India	To outline the critical facilitators and barriers that influence the implementation of telemedicine in the Indian health care system, observed at the infrastructural, sociocultural, regulatory, and financial levels, from the perspectives of health care providers, patients, patient caregivers, society, health organizations, and the government	Telemedicine	46	Analysis of the literature revealed key barriers such as data privacy and security concerns, physician and patient resistance to ICT, infrastructure issues, and ICT training gaps. Facilitators included reduced health care delivery costs, enhanced patient access to health care in remote areas, and shorter patient waiting times. The real-world experiences of Indian telemedicine practitioners and pioneers were also explored to complement literature-based perspectives on telemedicine implementation. Both stressed the need for reliable internet connectivity, technological adoption, comprehensive ICT training, positive sociocultural attitudes, stringent data privacy measures, and viable business models as being crucial to effective telemedicine adoption, with experts emphasizing practical adaptability alongside the literature-recognized facilitators.
[75]	Low- and middle-income countries (focus on China)	To comprehensively summarize the characteristics, barriers, and successful experiences in implementing telehealth services in China during the COVID-19 pandemic	Telemedicine	32	A total of 32 studies met the inclusion criteria. Successfully implementing and adopting telehealth in China during the COVID-19 pandemic necessitated strategic planning across aspects at society level (increasing public awareness and devising appropriate insurance policies); organizational level (training health care professionals, improving workflows, and decentralizing tasks); and technological level (strategic technological infrastructure development and designing inclusive telehealth systems). WeChat, a widely used social-networking platform, was the most common platform used for telehealth services. China’s practices in addressing the barriers may provide implications and evidence for other low- and middle-income countries with regard to implementing and adopting telehealth systems.

^a^SURE: supporting the use of research evidence.

^b^ICT: information and communications technology.

^c^mHealth: mobile health.

^d^TAM: Technology Acceptance Model.

^e^DOI: Diffusion of Innovations.

^f^DEDHI: Design and Evaluation of Digital Health Interventions.

^g^MOST: multiphase-optimization strategy.

^h^dHTA: digital health technology assessment.

#### Characteristics of Primary Research Within Reviews

Across the 18 SRs, there were 973 primary studies, with more than half of the studies (508/973, 52.2%) found in 3 (17%) SRs, each comprising 278 [58], 112 [59], and 118 [63] primary studies, respectively. The rest of the SRs included between 11 [70] and 50 [64] primary studies. Most of the SRs consisted of telemedicine-related primary research or studies with a digital health focus with relevance to telemedicine; however, the primary research in one SR [63] comprised only a small proportion (24/118, 20.3%) of telemedicine-focused primary studies, while the rest of the studies explored broader themes of telemedicine-related infrastructure, such as energy, and information and communication technology facilities. Duplication across the primary studies of the SRs was minimal, with only 16 (1.6%) of the primary studies found to be included in more than one SR (Multimedia Appendix 5 [58-75]). In addition, one SR included a systematic review primary study, which we also included in our umbrella review. Given the complex health systems research characteristics of the SRs, coupled with the limited overlap, it was deemed unfeasible to make additional adjustments to accommodate for this minor duplication within our analysis.

#### Channels, Clinical Specialties, and Methodological Approach

Analyzing in more detail the telemedicine channels, clinical specialties, and methodological approach of the SRs, considerable heterogeneity was found across reviews, although some SRs failed to analyze these aspects in their research (Multimedia Appendix 6 [58-75]). The main findings indicated that telemedicine channels were most frequently in the form of telephone calls and video consultations. Clinical specialties for telemedicine were largely focused on primary health care (such as management of chronic disease, weight loss, and smoking cessation). Methodological approaches used in the research predominantly comprised qualitative and mixed methods approaches, using both inductive and deductive synthesis, the latter drawing on a range of theoretical models to inform the analysis.

#### Country and Setting Characteristics

Analyzing the country focus of the SRs, 11 (61%) of the included studies took a global perspective in their reviews, while 6 (33%) focused on lower- and middle-income regional or country contexts, including China [75], India [74], the Middle East region [60], Africa [70], and Asia [63]. One review focused exclusively on the United States [68]. The country of origin of the research teams was diverse, with 7 (39%) studies comprising multicountry research teams. Regarding the country of origin of the first author, the United Kingdom and Iran dominated, each with 3 (17%) studies; followed by Switzerland and the United States with 2 (11%) studies each; and the rest (8/18, 44%) from Australia, Canada, Germany, India, Norway, Portugal, Spain, and South Africa. A somewhat balanced gender distribution was found across first authors based on an individual search of name and image, comprising 10 (56%) men and 8 (44%) women, although this should be interpreted with caution given the limitations of this approach. Interestingly, although there were no language restrictions on the search, all the studies screened were in English.

Not all the SRs synthesized the country of origin of primary studies. However, those that did demonstrated the broad spectrum of country contexts reflected across the primary research. Most of the SRs included primary studies either from multiple countries or focused on regional contexts [60,63,70], with the exception of the aforementioned ones that took a single country focus [68,74,75]. Many other SRs reported a high proportion of the primary research originating from the United States [58,59,62,64,66,71], with the United Kingdom, Australia, and Canada also frequently mentioned. The considerable global representation of the primary research across SRs comprises 63 countries, spanning the Americas, Europe, Africa, the Middle East, and Asia and the Pacific.

The setting or context of the primary research was synthesized in 11 (61%) of the included reviews, and there was considerable heterogeneity in this analysis. Where context was analyzed, most primary studies were reported to be located in a health facility, at tertiary, secondary, primary, or community level [63,65-67,69,73-75], with several indicating low-resource, rural, or remote health facility contexts [63,65-67,69,75]. Other settings included in the primary research comprised ambulatory and outpatient care [68], home-based care and self-care [63,66,67,69,71,73], and health system–wide studies [67,69,74].

#### Year of Publication

Regarding year of publication, defined by the state-of-the-art search filter (from 2018 to 2024), 4 (22%) of the SRs were published in 2023, followed by 2020, 2021, and 2024, each with 3 (17%) published studies. In total, 2 (11%) studies were published in both 2018 and 2022, and finally, 1 (6%) study was published in 2019. Conversely, the distribution of the 973 primary studies included within the SRs peaked at 216 (22.2%) in 2020, followed by 101 (10.4%) in 2015, and, close behind, 100 (10.3%) in 2016. Surprisingly, given publication lag times, there were also several (28/973, 2.9%) primary studies included from 2022 and 2023, although none from 2024, and very few (20/973, 2.1%) primary studies were published before 2005, with the earliest study dating back to 1998.

### Telemedicine Implementation Conceptual Lens

The SRs explored telemedicine implementation dimensions through different conceptual lenses. A case study approach was taken by half of the studies (9/18, 50%), either exploring implementation from a regional [60,63] or a country perspective [68,74,75], or an intervention, such as remote patient monitoring [71], telerehabilitation [73], virtual care [61], and home-based care [62], with one focusing on the COVID-19 pandemic [59]. In total, 3 (17%) of the reviews examined telemedicine implementation from the perspective of implementation constructs, such as trust [58], change management [66], and telemedicine infrastructure [63]. The remaining studies explored implementation theories, drawing on various models relating to design [67], implementation readiness and assessment [69,72], policy [70], spread and scale [65], and other theoretical models [64].

### KTs Compared

Each SR synthesized the findings of its study of the constructs of telemedicine implementation into a “KT” (Multimedia Appendix 7 [58-75] provides a summary of the constructs of each KT). KTs can be broadly grouped into 4 types. First, diagrammatic schemas of implementation provide conceptual models of telemedicine implementation [64-67,70,71]. Second, some (2/18, 11%) SRs consolidated and mapped the dimensions of existing theories [69,72]. Third, several KTs took the form of an implementation matrix delineating enablers and impediments [58,59,62,63,68,74,75]. Finally, 2 (11%) reviews provided a set of thematic strategic recommendations to guide the implementation process [60,61]. Many of the SRs emphasized that their respective tools had been designed to support implementation in practice [60,63-67,70-72,74], although none indicated that the tool had been field tested.

Comparing the telemedicine implementation constructs of the KTs to each other (Tables 2 and 3), there was a high degree of heterogeneity in the conceptualization of implementation constructs across KTs. The taxonomies of KTs varied across SRs, with most being poorly defined and lacking in definitional boundaries. Even those that, on the surface, appeared to have a degree of similarity [67,70] were, on closer analysis, conceptualized, defined, and delineated differently. However, 2 broad dimensions of implementation were apparent across the KTs: those relating to the “process or lifecycle” of telemedicine implementation, and those that were more “thematic” elements of implementation. Overall, there was a predominance of the latter approach, with 14 (78%) of the reviews exploring telemedicine implementation in a “snapshot” or fixed point [58-64,68-70,72-75], while the remaining 4 (22%) took a process or life cycle approach, exploring how implementation had evolved longitudinally as the telemedicine system matured [65-67,70].

**Table 2:** Comparison of the process constructs of telemedicine implementation knowledge tools.

References	Process dimensions				
	Readiness and needs assessment (n=10)	Road map and planning (n=9)	Managing change (n=7)	Implementing telemedicine services (n=17)	Continuous improvement and measuring performance (n=11)
[58]				✓	
[59]				✓	✓
[60]		✓		✓	✓
[61]		✓	✓	✓	
[62]			✓	✓	
[63]	✓			✓	
[64]	✓	✓	✓	✓	✓
[65]	✓	✓	✓	✓	✓
[66]	✓	✓	✓	✓	✓
[67]	✓	✓		✓	✓
[68]	✓		✓	✓	
[69]	✓				
[70]	✓	✓		✓	✓
[71]		✓		✓	✓
[72]	✓			✓	✓
[73]	✓	✓	✓	✓	✓
[74]				✓	
[75]				✓	✓

**Table 3:** Comparison of the thematic constructs of telemedicine implementation knowledge tools.

References	Thematic dimensions						
	Human and sociocultural aspects (n=18)	Organization, operations, management, and leadership (n=18)	Communication and coordination (n=7)	Policy, legal, and financial considerations (n=16)	Technical, technology, and infrastructure (n=18)	Clinical health condition and quality of care (n=14)	Wider context (n=11)
[58]	✓	✓			✓		
[59]	✓	✓		✓	✓	✓	
[60]	✓	✓		✓	✓		✓
[61]	✓	✓	✓	✓	✓	✓	
[62]	✓	✓	✓	✓	✓		✓
[63]	✓	✓		✓	✓		
[64]	✓	✓	✓	✓	✓	✓	
[65]	✓	✓		✓	✓	✓	✓
[66]	✓	✓	✓		✓	✓	
[67]	✓	✓		✓	✓	✓	✓
[68]	✓	✓		✓	✓	✓	
[69]	✓	✓		✓	✓	✓	✓
[70]	✓	✓		✓	✓	✓	✓
[71]	✓	✓	✓	✓	✓	✓	✓
[72]	✓	✓		✓	✓	✓	✓
[73]	✓	✓	✓	✓	✓	✓	✓
[74]	✓	✓		✓	✓	✓	✓
[75]	✓	✓	✓	✓	✓	✓	✓

Considering the telemedicine implementation life cycle, most attention across the KTs was focused on the actual point of implementation of telemedicine services, rather than before the implementation or optimization. In relation to the thematic dimensions of implementation, the human and social, organizational, technology and infrastructure, and legal policy and financial aspects of telemedicine services received the most attention. Interestingly, across the KTs there were limited attempts to quantitatively rank the relative importance of implementation constructs beyond anecdotal efforts, except for one review which found, using latent semantic analysis, that the highest correlation was in the “human” dimension, across the constructs of “legal,” “financial,” and “privacy” [70].

### Telemedicine Implementation Construct Analysis

#### Readiness and Needs Assessment

Upon closer examination of the process and thematic constructs of telemedicine implementation, the readiness or preparedness of the health system emerged as a recurrent theme across many of the SRs. For Mauco et al [69], readiness was a pivotal focus, emphasizing the importance of understanding the needs and preparedness of the ecosystem at the organizational, technological-infrastructural, health care provider, engagement, societal, government, and public-patient level, as well as the core preparedness of the community. Other reviews stressed the importance of understanding the “context,” particularly regarding the end users and their sociocultural characteristics, capacities, needs, digital literacy, available resources, perceptions, clinical conditions, and access to technological infrastructure, as well as the digital divide [59,60,62,63,71,75]. The importance of understanding equity issues of the context was also highlighted [59]. Government readiness was a recurring theme, especially regarding adequate financial resources and favorable regulatory conditions [60,62,63,67,69]. Another important finding was the need for preparedness and understanding of the telemedicine technological and infrastructure context [63,65,66,70]. Interestingly, however, only a couple of SRs (2/18, 11%) specifically stated the importance of conducting a needs assessment or situational analysis [66,69], while 1 (6%) SR emphasized the importance of having the right “evidence base” for the intervention [73].

#### Road Map and Planning

Several of the SRs emphasized the importance of having a strategic plan, road map, or at least an operational plan for telemedicine implementation, identifying it as a key success factor. The need to create clear, common, and simple telemedicine vision, mission, values, and priorities was highlighted [66,70], as was the importance of developing a telemedicine strategic plan [60,65-67,75], which others articulated as ensuring the right “value proposition” [65]. A telemedicine strategy was found to facilitate the allocation of resources, organizational change, and local action planning and helped to guide monitoring and evaluation of telemedicine services [60]. The importance of engaging different telemedicine stakeholders in strategy development was found to strengthen the tailoring of the telemedicine vision to their needs and realities, while supporting “buy-in” and commitment [66]. There were limited findings in the SRs on strategy content, although areas of focus included integration within the wider health system and interoperability [60,67,70]; technological considerations [75]; legislation, governance, and financial considerations [70,75]; training plans [70,73]; and quality-of-care preparedness [70]. The findings of Kho et al [66] highlighted that primary studies often focus on either “strategy” or “operational” factors and that a lack of balance between the 2 compromised implementation success.

#### Managing Change

A recurring theme across the SRs was the importance of managing change, commonly understood as the processes and techniques used to support the human aspects of achieving desired organizational outcomes [76]. One study [66] was entirely dedicated to change management, emphasizing the importance of building trust and the use of change management theory to support implementation processes and celebrating success. Other reviews highlighted the need for structural change and the adoption of change management practices and principles [59-62,64,65,68,70]. Specific changes that were stressed included change of organizational and clinical protocols; workflows and role definitions [60,61,64,65,68,70,73]; the need for supportive leadership, management, and change champions [59,61,65,68,70,75]; organizational integration and interoperability [59,70]; good internal and external communications especially to support policy change [60,62,65,68,73]; changes associated with incentives and financial reimbursements [62,68]; training to support change [60,64,65,68,75]; and end-user behavior change and engagement [68,70,71,73,75]. The opportunity that the COVID-19 pandemic provided as a driver of rapid and decisive change for global telemedicine scale-up was also highlighted [68]. Resistance to change was identified as one of the biggest barriers to telemedicine implementation [60,66,73], and to address this, SRs stressed the importance of communicating the need for change, the degree of “fit,” the advantages and relevance of telemedicine, along with reinforcing job security, autonomy, and empowerment for health providers [64]. The scale-up and sustainability of telemedicine services were identified as being directly related to the ability of the organization to manage change, and the frequent underestimation of the degree of change was also stressed [65]. Moreover, interdependencies and relational aspects of the change process were found to support the necessary sense-making and experimentation required to enable telemedicine to be adapted to different contexts [65].

#### Implementing Telemedicine Services

Across the SRs, the design, development, and deployment of telemedicine—both at the health facility level and within the broader community—received the most attention. In relation to this aspect, the end users (both health providers and patients) were a major focus, with emphasis on capacity building, training, digital literacy, and confidence building [58,60-63,66,70,71,73,75], as well as building trust [58,63,66] and buy-in [64,65,75]. Organizational and operational aspects were also stressed, particularly the role of management, leadership, and strong execution [60,66]. Communication, coordination, and collaboration across diverse stakeholders were identified as challenging yet essential [61,62,64,66,71,73,74]. Much attention was given to operationalizing evidence-based clinical guidelines and protocols [58,60,66,68] and patient-centered care, quality of care, and clinical safety [59,71,72,75]. Local financial considerations were also highlighted in the SRs to ensure adequate resourcing of telemedicine services at facilities [60,62,64,66-69,75]. However, technology- and infrastructure-related issues received the most attention across the SRs, particularly the need to ensure adequate technological infrastructure for end users, including access to a helpdesk [59-63,65,70,71,73], as well as human-centered design and user-friendly technology [58,59,62,72,73]. To best support this, it was suggested, wherever possible, to use devices and platforms that end users are already familiar with [62]. The importance of operationalizing regulations and policy, particularly around issues of privacy, security, and confidentiality of data [62,67,70], was also highlighted. Surprisingly, however, only 1 (6%) review mentioned the importance of ensuring informed consent [62].

#### Continuous Improvement and Measuring Performance

The ongoing review of telemedicine implementation performance was also identified as critical to the success of telemedicine services. The findings suggest that a mix of regular and periodic data collection on telemedicine implementation and performance was needed to support continuous improvement and sharing of lessons learnt [66,73,75], thus facilitating an iterative, flexible, and agile approach to telemedicine implementation to support trial and error [66,70,73]. A user-friendly data visualization dashboard was found to be helpful for decision makers [71]. Various performance indicators were identified, including patient outcomes comprising a mix of admission and readmission data, emergency department visits, access and equity monitoring, changes in patterns of health service use, quality of life, user experience, privacy, and security [59,66,67,71]. Other indicators identified included patient and health care provider satisfaction [66,67], service quality and performance improvement [60,67,70], workflow output reviews [66], and cost-effectiveness analysis [60,66,71]. The issue of poor-quality data in low-resource, low-tech contexts was identified as a potential risk to telemedicine implementation and practice [60].

## Discussion

### Principal Findings

By conducting evidence synthesis of implementation theories, models, and frameworks, this umbrella review provides a global perspective of the state-of-the-art of telemedicine implementation constructs. The findings represent 63 countries comprising low- to high-resource contexts and a range of settings from clinical care to self-care. Areas of convergence and discrepancies in the conceptualization of implementation constructs are identified, thereby contributing to the urgent need for consensus building on the dimensions of telemedicine implementation to support knowledge transfer and guide evidence-based high-quality telemedicine implementation. The findings of our evidence synthesis highlight the state-of-the-art essential requirements for a national telemedicine implementation ecosystem, structured into 2 overarching categories: process and thematic dimensions. The process dimensions include readiness and needs assessment, road map development and planning, change management, implementation of telemedicine services, and continuous improvement and performance measurement. The thematic dimensions cover human and sociocultural factors; organization, operations, management, and leadership; communication and coordination; policy, legal, and financial considerations; clinical conditions and quality of care; and the broader contextual environment.

Comparing our findings to those obtained from similar studies, none of the latter studies were found to be directly comparable in undertaking an umbrella review of telemedicine implementation constructs. The closest study is an umbrella review conducted by Mair et al [77] and later updated by Ross et al [78] focusing on digital health rather than on the unique characteristics of telemedicine architecture. However, similar to our study, those studies identified the multiple levels and factors to be considered in digital health implementation, including technological considerations, organizational issues, health professionals, and the processes of implementation. Some other relevant studies were either limited in scope, focusing on a single stage or aspect of telemedicine implementation, such as needs assessment, readiness and evaluation [79-83], and end-user experience [84], or were outdated given this rapidly evolving field [33,85,86]. Others [82,87] identified that implementation KTs varied in their degree of robust development and were often characterized by limitations such as a lack of evidence base and expert consultation; weak theoretical foundations; and, similar to our study, found limited field testing of KTs, an absence of practical guidance and evaluation, and excessive complexity regarding their application in practice.

A KT that incorporates most of the telemedicine implementation constructs identified in this study and attempts to address the limitations identified in other telemedicine implementation tools is the support tool to strengthen telemedicine: guidance for telemedicine assessment and strategy development, developed by the WHO [88]. This KT comprises a succinct evidence-based tool to support knowledge translation and guide practice. A unique strength of this tool is its conceptual architecture, which identifies telemedicine implementation cores (process indicators), domains (thematic dimensions of implementation), and items (implementation activities). The tool is informed by evidence synthesis [3] and a Delphi consensus study, the aim of which was to align expert opinion on telemedicine implementation constructs [89], and is currently being field-tested in various countries. This knowledge translation tool is not context, disease, user group, or setting specific; rather, it provides a generic resource that describes the building blocks required for the development of a successful telemedicine service along the life cycle of telemedicine maturity, which is adaptable to local needs and available resources.

A critical finding from our study is the importance of telemedicine implementation KTs that are flexible, enabling them to be adapted to different contexts and to support iterative, continuous learning. A recurring theme is the need for KTs to be adaptable to the realities of different cultural, regulatory, funding, capacity, and resourcing levels, particularly in the diverse contexts of lower- and middle-income countries. To support this, our study identified the substantial alignment of experts on the essential constructs of telemedicine implementation despite the considerable heterogeneity in the conceptualization of KTs. Finally, although boundaries of telemedicine implementation constructs are fuzzy and relationships across them are dynamic, this study emphasizes the operational imperative of delineating and defining constructs to help support the allocation of roles and responsibilities, resourcing, and coordination and collaboration for telemedicine implementation actors.

### Strengths and Limitations

This study has both strengths and limitations. A strength of the study is its timely contribution to a complex, urgent gap in knowledge translation, which is hindering the global implementation and scale-up of sustainable, high-quality telemedicine systems. By synthesizing the state-of-the-art evidence of KTs, this study contributes to informing the development of meaningful KTs that support the pressing needs of practitioners who are designing, developing, and optimizing telemedicine services. The limitations of the study were mentioned earlier and mostly consist of modifications associated with accelerating the study methodology in accordance with “rapid review” guidance, implying a degree of caution in interpreting the results while enabling timeliness in sharing the findings.

### Conclusions

This study contributes to an urgent real-world public health problem by synthesizing state-of-the-art evidence of telemedicine implementation to inform knowledge translation, thereby furthering the implementation of high-quality, sustainable, and scalable telemedicine systems. An important finding from this study is the need to continue building consensus on what the essential evidence-based telemedicine implementation constructs are, while providing flexibility to enable implementation to be adapted to the needs and realities of different contexts. Future implementation research needs to focus on field-testing evidence-based KTs for usability and adaptability to different telemedicine implementation settings.

## Appendix

Multimedia Appendix 1 PROSPERO research protocol.

Multimedia Appendix 2 Search strategy.

Multimedia Appendix 3 Extraction table protocol.

Multimedia Appendix 4 Critical appraisal of the included reviews.

Multimedia Appendix 5 Duplication of primary research across the included reviews.

Multimedia Appendix 6 Other characteristics of the included reviews.

Multimedia Appendix 7 Summary of knowledge tool outputs of the included reviews.

Multimedia Appendix 8 PRISMA checklist.

## Abbreviations

KT: knowledge tool
MeSH: Medical Subject Headings
PRISMA: Preferred Reporting Items for Systematic Reviews and Meta-Analyses
SR: systematic and scoping reviews
WHO: World Health Organization
