# Supplementary material for: The State of the Art of Telemedicine Implementation Architecture: Rapid Umbrella Review of Systematic Reviews
Source: J Med Internet Res. 2025 Jun 9;27:e70276. doi: 10.2196/70276 (PMC12186003; doi:10.2196/70276)
Supplement: Multimedia Appendix 1 [file jmir_v27i1e70276_app1.pdf]

NIHR | National Institute for Health Research

PROSPERO
International prospective register of systematic reviews

State of the art of the Telemedicine Implementation Architecture: A Rapid Umbrella Review of Systematic Reviews

To enable PROSPERO to focus on COVID-19 submissions, this registration record has undergone basic automated checks for eligibility and is published exactly as submitted. PROSPERO has never provided peer review, and usual checking by the PROSPERO team does not endorse content. Therefore, automatically published records should be treated as any other PROSPERO registration. Further detail is provided here.

Citation

Che Katz, José Maria Ruiz Serrats, Francesc Saigí Rubió. State of the art of the Telemedicine Implementation Architecture: A Rapid Umbrella Review of Systematic Reviews. PROSPERO 2024 CRD42024512516 Available from: https://www.crd.york.ac.uk/prospero/display_record.php?ID=CRD42024512516

Review question

• What is the state-of-the-art of the implementation constructs of the telemedicine architecture?

• What does the evidence suggest about the determinants, mechanisms, domains, and building blocks of implementing a telemedicine service?

• What constructs have been found to be most important in the design, development, deployment, and optimization of telemedicine interventions?

• What are the recommendations about the telemedicine implementation architecture which can inform knowledge translation tools (ie guidelines, frameworks maturity models) for telemedicine deployment?

Searches

Three databases will be searched: PubMed, Web of Sciences, and Scopus.

Where feasible this will be supplemented by additional searches in (i) systematic review suppositories (ie JBI Evidence Synthesis, Cochrane Evidence Database, Prospero, DARE, Epistemonikos, and Campbell Collection), (ii) reviewing automated recommendations from search engines of relevant reviews, (iii) recommendations from telemedicine experts and (iv) back and forward searches of selected sources.

Types of study to be included

Only systematic reviews demonstrating reproducible methods and a systematized search strategy will be included in the study. Within the systematic review study category, all study designs will be considered eligible, including quantitative, qualitative, and mixed-method studies.

Condition or domain being studied

The research examines telemedicine implementation determinants and constructs through an implementation science research lens, to further knowledge translation of evidence-based telemedicine implementation. The research focusses on implementation of all types of telemedicine interventions, at all levels of the health system, with any disease target, setting, populations, or context.

Participants/population

A population of focus is not specified in this study, rather the research explores all types of telemedicine services (irrespective of user group) from the perspective of organizational structures at macro, meso and micro level (i.e from health system to health facility level) examining the evidence base for telemedicine implementation determinants, constructs, mechanisms, processes, and practices.

Intervention(s), exposure(s)

Inclusion: Explores constructs / architecture of telemedicine/telehealth implementation at scale (ie design, development, maturity models, readiness, needs assessments, guidelines, models, frameworks and other tools.)

Exclusion: Not related to telemedicine/telehealth implementation. Does not explore constructs/architecture of telemedicine/telehealth implementation. Primarily focusses on telemedicine clinical outcomes, health system outcomes, and/or evaluation of telemedicine services. Constructs of implementation of other interventions (such as digital health, eHealth mHealth, uHealth, AI, robotics, digital health etc), with no inclusion of telemedicine.

Comparator(s)/control

The study will consider any type of comparator or control group, including the comparison with traditional care/face-to-face patient care. Studies without a control group will also be eligible for inclusion

Context

There is now a strong evidence base for the global scale-up of telemedicine services, however the implementation of telemedicine is recognized to be a complex multi-level multi-faceted innovation; with experts drawing attention to the gap between the vision and practice. This study examines the evidence base for the building blocks, or architecture, of a telemedicine implementation including readiness, strategy, the end-users (both health providers and consumers), leaders and managers, organizational structures, clinical and economic considerations, technical and infrastructure, and regulatory and ethical aspects. To the best of our knowledge no previous umbrella review has been collated and comprehensively summarized data from multiple studies about the constructs of telemedicine implementation. Therefore, the aim of this systematic review is to synthesize the global evidence of telemedicine implementation, particularly in consideration of the recent global expansion of telemedicine services since the Covid-19 pandemic. The results of this study will support furthering the quality, scale-up, sustainability, and knowledge translation of evidence-based telemedicine implementation know-how and tools.

Main outcome(s)

Outcomes of the study will be assessed building on identified telemedicine implementation determinants in the broad domains of individual, organization, clinical, economic, technological, regulations and monitoring and evaluation. These will be mapped against relevant models and theories drawn from the domains of implementation science research including consideration of implementation outcomes such as acceptability, adoption, appropriateness, cost, feasibility, fidelity, penetration, and sustainability. As the study will be examining knowledge translation tools (guidelines, models, frameworks, readiness assessment, maturity models etc), other outcomes frameworks that explore contextual factors may also be applied. The study will seek to identify an evidence-based set of telemedicine implementation constructs and mechanisms that will subsequently inform the development of a Support tool to strengthen telemedicine: guidance for telemedicine assessment and strategy development for the World Health Organization Regional Office for Europe (WHO/Europe).

Measures of effect

It is anticipated that each systematic review included will describe the characteristics of the measure effect of the studies in their own extraction tables, and synthesis these in their analysis. This study will further summarize and synthesize the included systematic reviews in an extraction table, incorporating measure effect, and further synthesis these in the analysis.

Additional outcome(s)

None

Measures of effect

Not applicable

Analysis is anticipated to be primarily qualitative combining a meta-aggregative approach with narrative, thematic and framework synthesis.

Data extraction (selection and coding)

All studies sourced will be uploaded to Covidence and de-duplicated. Two researchers will screen each study independently (title and abstract) based on the inclusion and exclusion criteria. Interrater conflicts will be resolved by a third researcher and/or further discussion. A PRISMA flow chart will summarize the search process. Data extraction will be undertaken according to the data extraction format. Data to be extracted will include study ID, first author, year of publication, country where the study was carried out, study design, aim of the study, telemedicine type, sample size, and findings.

Risk of bias (quality) assessment

The relevance of a critical appraisal will be determined according to the characteristics of the reviews retrieved, as recommended by the WHO guidance Rapid reviews to strengthen health policy and systems: a practical guide. then-health-policy-and-systems-a-practical-guide (Tricco AC, Langlois EV, & Straus SE (Eds.), 2017) https://ahpsr.who.int/publications/i/item/2017-08-10-rapid-reviews-to-strengthen-health-policy-and-systems-a-practical-guide. If deemed necessary, an appraisal tool will be selected which is practical and relevant (ie JBI Critical appraisal for checklist for systematic reviews and research synthesis, AMSTAR2)

Strategy for data synthesis

A qualitative synthesis will be undertaken, which includes reporting on the results and combining results from studies into a matrix map of implementation constructs, to identify common characteristics and exceptions.

Analysis of subgroups or subsets

None

Contact details for further information

Che Katz

ckatz@uoc.edu

Organisational affiliation of the review

Universitat Oberta de Catalunya

https://www.uoc.edu/portal/en/estudis_arees/ciencies-salut/index.html

Review team members and their organisational affiliations

Ms Che Katz. Universitat Oberta de Catalunya

Mr José Maria Ruiz Serrats. Universitat Oberta de Catalunya

Assistant/Associate Professor Francesc Saigí Rubió. Universitat Oberta de Catalunya

Type and method of review

Intervention, Narrative synthesis, Review of reviews, Service delivery, Synthesis of qualitative studies, Systematic review, Other

Anticipated or actual start date

14 February 2024

Anticipated completion date

31 August 2024

Funding sources/sponsors

None

Grant number(s)

State the funder, grant or award number and the date of award

None

Conflicts of interest

Language

English

Country

Spain, Ukraine

Stage of review

Review Ongoing

Subject index terms status

Subject indexing assigned by CRD

Subject index terms

MeSH headings have not been applied to this record

Date of registration in PROSPERO

24 February 2024

Date of first submission

13 February 2024

Details of any existing review of the same topic by the same authors

There are no pre-existing reviews on this topic undertaken by the authors of this review.

Stage of review at time of this submission

The review has not started

Stage	Started	Completed
Preliminary searches	No	No
Piloting of the study selection process	No	No
Formal screening of search results against eligibility criteria	No	No
Data extraction	No	No
Risk of bias (quality) assessment	No	No
Data analysis	No	No

The record owner confirms that the information they have supplied for this submission is accurate and complete and they understand that deliberate provision of inaccurate information or omission of data may be construed as scientific misconduct.

The record owner confirms that they will update the status of the review when it is completed and will add publication details in due course.

Versions

24 February 2024

24 February 2024
