# Supplementary material for: The State of the Art of Telemedicine Implementation Architecture: Rapid Umbrella Review of Systematic Reviews
Source: J Med Internet Res. 2025 Jun 9;27:e70276. doi: 10.2196/70276 (PMC12186003; doi:10.2196/70276)
Supplement: Multimedia Appendix 2 [file jmir_v27i1e70276_app2.docx]

**Multimedia Appendix 2 - Search Strategy**

On 20 May 2024 a search was undertaken in three databases PubMed, Web of Science, and Scopus, with the following search strategy.

| #1 | telemedicine OR telecare OR telehealth OR “remote consultation” OR e-Health OR eHealth OR “digital health” [Title/Abstract]  AND  telemedicine OR telehealth OR telecare [PubMed=Text Word. Web of Science/Scopus=All fields] |
| --- | --- |
| #2 | implementation OR intervention OR execution [Title/Abstract]  NOT  evaluation OR performance OR outcome [Title/Abstract] |
| #3 | tool* OR guide* OR model OR framework [Title/Abstract] |
| #4 | “systematic review” OR “scoping review” [Title/Abstract] |
| #5 | #1 AND #2 AND #3 AND #4 |
|  | Filter 2018 to 2024 |

The search string for PubMed, which was indicative of the other searches, was as follows:

((((telemedicine[Title/Abstract] OR telecare[Title/Abstract] OR telehealth[Title/Abstract] OR "remote consultation"[Title/Abstract] OR e-Health[Title/Abstract] OR eHealth[Title/Abstract] OR "digital health"[Title/Abstract]) AND (telemedicine[Text Word] OR telehealth[Text Word] OR telecare[Text Word])) AND ((implementation[Title/Abstract] OR intervention[Title/Abstract] OR execution[Title/Abstract]) NOT (evaluation[Title/Abstract] OR performance[Title/Abstract] OR outcome[Title/Abstract]))) AND (tool*[Title/Abstract] OR guide*[Title/Abstract] OR model[Title/Abstract] OR framework[Title/Abstract])) AND ("systematic review"[Title/Abstract] OR "scoping review"[Title/Abstract]) Filters: from 2018 - 2024 Sort by: First Author
