# Supplementary material for: The State of the Art of Telemedicine Implementation Architecture: Rapid Umbrella Review of Systematic Reviews
Source: J Med Internet Res. 2025 Jun 9;27:e70276. doi: 10.2196/70276 (PMC12186003; doi:10.2196/70276)
Supplement: Multimedia Appendix 3 [file jmir_v27i1e70276_app3.docx]

**Multimedia Appendix 3 – Extraction Table Protocol**

| **Variable to be extracted** | Explanation | Type of extraction |
| --- | --- | --- |
| **Systematic Review citation** | |  |
| **Last Name** | The first author’s last name | Verbatim |
| **Year of Publication** | Year of publication as indicated in the citation | Verbatim |
| **Title** | Title of paper as indicated in the citation. | Verbatim |
| **Journal Name/Volume** | Journal name and volume as indicated in the citation | Verbatim |
| **Characteristics of the Systematic Review** | |  |
| **Abstract** | As indicated in the paper | Verbatim |
| **Focus: Telemedicine / Digital Health** | Determine the main focus of the SR -  Telemedicine (defined as any form of telehealth, telecare etc)  Digital Health (defined as broader in scope than only telemedicine) | Own words |
| **Country:  Author Origin** | Insert the country of authors in order of placement | Verbatim |
| **Country Study Focus** | Insert the country/region of focus of the SR (ie is the paper applied to a specific country/region or is it global in nature). | Own words |
| **Study Methodology** | State the type of evidence synthesis (ie systematic review, systematic literature review, scoping review) – usually stated in the title | Verbatim |
| **Purpose of Study** | The objective of the SR. | Verbatim |
| **Research Questions** | The study questions if specified | Verbatim |
| **Range (years) of included studies** | Indicate the duration of the search filter for the SR. If the whole year is applied, no need to include the month. | Verbatim |
| **Databases Searched** | Describe which databases were used for the search. | Verbatim (though some editing for consistency) |
| **Methodology of Analysis** | Describe the methodology of analysis/synthesis used by the SR (ie is it more qualitative, quantitative, mixed method, inductive/deductive etc). | Own words |
| **Ethical approval** | Is ethical approval indicated  (Yes/No/Not indicated) | Own words |
| **Characteristics of the primary studies within the SR** | |  |
| **Number of primary studies included** | Describe the number of included primary studies considered in the review, usually indicated in the PRISMA. | Verbatim |
| **Years of Primary studies included** | List the year (number of primary studies) (ie 2004 (2), 2022 (1). If this is not stated in the systematic review, this should be synthesized from the extraction table. | Verbatim (or own words if not provided) |
| **Methodology of studies included** | List the primary study methodological designs considered in the SR. If this is not stated in the SR, this should be synthesized from the extraction table. If not available in the extraction table state ‘not available’. | Verbatim (or own words if not provided) |
| **Country of Origin of included studies** | List the country of origin of included primary studies. If this is not stated in the SR, this should be synthesized from the extraction table. If not available in the extraction table state ‘not available’. | Verbatim (or own words if not provided) |
| **Settings/Context** | Briefly describe the settings/scenarios primarily focused on the primary studies. (ie rural, urban, health facility, health system wide). If this is not stated in the SR, this should be synthesized from the extraction table. If not available in the extraction table state ‘not available’. | Verbatim (or own words if not provided) |
| **Type of telemedicine interventions included** | Insert the telemedicine specialties considered in the manuscript. Wider digital health focused SRs may include other digital health applications, however for this study only telemedicine related interventions are of interest. If this is not stated in the SR, this should be synthesized from the extraction table. If not available in the extraction table state ‘not available’. | Verbatim (or own words if not provided) |
| **Type of clinical specialties included** | Insert the clinical specialties considered in the manuscript. If this is not stated in the SR, this should be synthesized from the extraction table. If not available in the extraction table state ‘not available’. | Verbatim (or own words if not provided) |
| **Theoretical models/frameworks** | Insert a summary of any theoretical models that were synthesized from the primary studies. If this is not stated in the SR, this should be synthesized from the extraction table. If not available in the extraction table state ‘not available’. | Verbatim (or own words if not provided) |
| **Findings of the Systematic Reviews** | |  |
| **Theoretical Framework Underpinnings** | To analyze the implementation constructs several of the papers use a theoretical model or framework to map/code the data. Here the model should be stated in full (ie Consolidated Framework for Implementation Research) | Verbatim (or own words if not provided) |
| **Findings** | List the main findings highlighted in the study. This might be a little bit challenging, as some studies tend to have an extensive result section. Only include data that will attract the future reader and give knowledge to healthcare providers, policymakers, and the scientific community. | Paraphrase  /own words |
| **Recommendations** | List the main recommendations highlighted in the study. Only include data that will attract the future reader and give knowledge to healthcare providers, policymakers, and the scientific community. | Paraphrase  /own words |
| **Knowledge Tool Output** | State the title and main characteristics of the knowledge tool output produced from the study. | Paraphrase  /own words |
| **Limitation of the study** | List the manuscript limitations often stated at the end of the paper. | Paraphrase  /own words |
| **Critical Appraisal** | |  |
| **JBI Critical Appraisal Checklist for Systematic Reviews and Research Syntheses** | Insert the final score of the critical appraisal |  |
