# Supplementary material for: The State of the Art of Telemedicine Implementation Architecture: Rapid Umbrella Review of Systematic Reviews
Source: J Med Internet Res. 2025 Jun 9;27:e70276. doi: 10.2196/70276 (PMC12186003; doi:10.2196/70276)
Supplement: Multimedia Appendix 4 [file jmir_v27i1e70276_app4.pdf]

Multimedia Appendix 4 - Critical appraisal of included reviews

Systematic Review Author	1. Is the review question or objective stated?	2. Were the inclusion criteria appropriate for the review question?	3. Was the search strategy appropriate?	4. Were the sources and resources used to search for studies adequate?	5. Were the criteria for appraising studies appropriate?	6. Was critical appraisal conducted by two or more reviewers independently?	7. Were there methods to minimize errors in data extraction?	8. Were the methods used to combine studies appropriate?	9. Was the likelihood of publication bias assessed?	10. Were recommendations for policy and/or practice supported by the reported data?	11. Were the specific directives for new research appropriate?	Score: 1 - meets all the criteria / Very Good 2 - meets most of the requirements /Good 3 - methodological weaknesses / Fair 4 - consider rejecting / Poor	DECISION
Adjekum et al. (2018)	Y	Y	Y	Y	N	N	N	P	Y	Y	Y	3 (Fair)	Include
Alipour and Hayavi-Haghighi (2021)	Y	Y	Y	Y	Y	Y	Y	Y	Y	Y	N	2 (Good)	Include
Al-Samarraie et al. (2020)	Y	Y	Y	Y	P	Y	Y	Y	Y	Y	N	2 (Good)	Include
Babaei et al. (2023)	Y	P	Y	Y	N	N	N	P	P	Y	Y	3 (Fair)	Include
Galavi et al. (2022)	Y	Y	Y	Y	Y	U	P	Y	P	Y	NA	2 (Good)	Include
Hui et al. (2022)	Y	Y	Y	Y	P	N	P	P	Y	Y	NA	2 (Good)	Include
Jacob et al. (2020)	Y	P	Y	Y	Y	Y	U	Y	Y	Y	Y	2 (Good)	Include
James et al. (2021)	Y	Y	Y	Y	Y	U	U	Y	Y	Y	Y	2 (Good)	Include
Kho et al. (2020)	Y	Y	Y	Y	NA	NA	P	Y	Y	Y	Y	2 (Good)	Include
Kowatsch et al. (2019)	Y	Y	Y	Y	N	NA	Y	Y	Y	Y	Y	2 (Good)	Include
Lieneck et al. (2021)	Y	Y	Y	Y	Y	Y	Y	P	Y	Y	Y	2 (Good)	Include
Mauco et al. (2018)	Y	P	P	Y	U	U	U	Y	U	Y	Y	3 (Fair)	Include
Mengiste et al. (2023)	Y	Y	Y	Y	P	P	Y	Y	Y	Y	Y	2 (Good)	Include
Miranda et al. (2023)	Y	Y	Y	Y	P	P	P	Y	P	Y	Y	2 (Good)	Include
Segur-Ferrer et al. (2024)	Y	Y	Y	Y	Y	N	N	Y	Y	Y	Y	2 (Good)	Include
Stampa et al. (2024)	Y	Y	Y	Y	N	P	Y	Y	Y	Y	Y	2 (Good)	Include
Venkataraman et al. (2024)	Y	Y	Y	Y	N	P	P	P	Y	Y	Y	2 (Good)	Include
Ye et al. (2023)	Y	Y	Y	Y	N	P	P	Y	Y	Y	Y	2 (Good)	Include

KEY: Y=Yes, N=No, P=Partially, U=Unclear, NA=Not Applicable

References

Adjekum, A., Blasimme, A., & Vayena, E. (2018). Elements of trust in digital health systems: Scoping review. *Journal of Medical Internet Research*, *20*(12), e11254. https://doi.org/10.2196/11254

Alipour, J., & Hayavi-Haghighi, M. H. (2021). Opportunities and Challenges of Telehealth in Disease Management during COVID-19 Pandemic: A Scoping Review. *Applied Clinical Informatics*, *12*(4), 864–876. https://doi.org/10.1055/s-0041-1735181

Al-Samarraie, H., Ghazal, S., Alzahrani, A. I., & Moody, L. (2020). Telemedicine in Middle Eastern countries: Progress, barriers, and policy recommendations. *International Journal of Medical Informatics*, *141*, 104232. https://doi.org/10.1016/j.ijmedinf.2020.104232

Babaei, N., Zamanzadeh, V., Valizadeh, L., Lotfi, M., Samad-Soltani, T., Kousha, A., & Avazeh, M. (2023). A scoping review of virtual care in the health system: infrastructures, barriers, and facilitators. *Home Health Care Services Quarterly*, *42*(2), 69–97. https://doi.org/10.1080/01621424.2023.2166888

Galavi, Z., Montazeri, M., & Ahmadian, L. (2022). Barriers and challenges of using health information technology in home care: A systematic review. *International Journal of Health Planning and Management*, *37*(5), 2542–2568. https://doi.org/10.1002/hpm.3492

Hui, C. Y., Abdulla, A., Ahmed, Z., Goel, H., Habib, G. M. M., Hock, T. T., Khandakr, P., Mahmood, H., Nautiyal, A., Nurmansyah, M., Panwar, S., Patil, R., Rinawan, F. R., Salim, H., Satav, A., Shah, J. N., Shukla, A., Tanim, C. Z. H., Balharry, D., & Pinnock, H. (2022). Mapping national information and communication technology (ICT) infrastructure to the requirements of potential digital health interventions in low and middle-income countries. *Journal of Global Health*, *12*, 04094. https://doi.org/10.7189/jogh.12.04094

Jacob, C., Sanchez-Vazquez, A., & Ivory, C. (2020). Understanding clinicians' adoption of mobile health tools: A qualitative review of the most used frameworks. *JMIR MHealth and UHealth*, *8*(7), e18072. https://doi.org/10.2196/18072

James, H. M., Papoutsi, C., Wherton, J., Greenhalgh, T., & Shaw, S. E. (2021). Spread, Scale-up, and Sustainability of Video Consulting in Health Care: Systematic Review and Synthesis Guided by the NASSS Framework. *Journal of Medical Internet Research*, *23*(1). https://doi.org/10.2196/23775

Kho, J., Gillespie, N., & Martin-Khan, M. (2020). A systematic scoping review of change management practices used for telemedicine service implementations. *BMC Health Services Research*, *20*(1), 815. https://doi.org/10.1186/s12913-020-05657-w

Kowatsch, T., Otto, L., Harperink, S., Cotti, A., & Schlieter, H. (2019). A design and evaluation framework for digital health interventions. *IT - Information Technology*, *61*(5–6), 253–263. https://doi.org/10.1515/ITIT-2019-0019

Lieneck, C., Weaver, E., & Maryon, T. (2021). Outpatient telehealth implementation in the united states during the covid-19 global pandemic: A systematic review. *Medicina (Lithuania)*, *57*(5), 462. https://doi.org/10.3390/medicina57050462

Mauco, K. L., Scott, R. E., & Mars, M. (2018). Critical analysis of e-health readiness assessment frameworks: suitability for application in developing countries. *Journal of Telemedicine and Telecare*, *24*(2), 110–117. https://doi.org/10.1177/1357633X16686548

Mengiste, S. A., Antypas, K., Johannessen, M. R., Klein, J., & Kazemi, G. (2023). eHealth policy framework in Low and Lower Middle-Income Countries; a PRISMA systematic review and analysis. *BMC Health Services Research*, *23*(1), 328. https://doi.org/10.1186/s12913-023-09325-7

Miranda, R., Oliveira, M. D., Nicola, P., Baptista, F. M., & Albuquerque, I. (2023). Towards A Framework for Implementing Remote Patient Monitoring From an Integrated Care Perspective: A Scoping Review. *International Journal of Health Policy and Management*, *12*(1), 7299. https://doi.org/10.34172/ijhpm.2023.7299

Segur-Ferrer, J., Moltó-Puigmartí, C., Pastells-Peiró, R., & Vivanco-Hidalgo, R. M. (2024). Methodological Frameworks and Dimensions to Be Considered in Digital Health Technology Assessment: Scoping Review and Thematic Analysis. *Journal of Medical Internet Research*, *26*(1), e48694. https://doi.org/10.2196/48694

Stampa, S., Thienel, C., Tokgöz, P., Razum, O., & Dockweiler, C. (2024). Factors Facilitating and Inhibiting the Implementation of Telerehabilitation—A Scoping Review. *Healthcare (Switzerland)*, *12*(6), 619. https://doi.org/10.3390/healthcare12060619

Venkataraman, A., Fatma, N., Edirippulige, S., & Ramamohan, V. (2024). Facilitators and Barriers for Telemedicine Systems in India from Multiple Stakeholder Perspectives and Settings: A Systematic Review. *Https://Home.Liebertpub.Com/Tmj*, *30*(5), 1341–1356. https://doi.org/10.1089/tmj.2023.0297

Ye, J., He, L., & Beestrum, M. (2023). Implications for implementation and adoption of telehealth in developing countries: a systematic review of China’s practices and experiences. *Npj Digital Medicine*, *6*(1), 174. https://doi.org/10.1038/s41746-023-00908-6
