# Supplementary material for: The State of the Art of Telemedicine Implementation Architecture: Rapid Umbrella Review of Systematic Reviews
Source: J Med Internet Res. 2025 Jun 9;27:e70276. doi: 10.2196/70276 (PMC12186003; doi:10.2196/70276)
Supplement: Multimedia Appendix 5 [file jmir_v27i1e70276_app5.pdf]

Multimedia Appendix 5 - Duplication of Primary Research across Systematic Reviews

		Systematic Review Author												
**Systematic Review Author**	**Duplicated Primary Studies**	**Adjekum et al. (2018)**	**Alipour and Hayavi-Haghighi (2021)**	**Galavi et al. (2022)**	**Jacob et al. (2020)**	**James et al. (2021)**	**Kho et al. (2020)**	**Lieneck et al. (2021)**	**Mengiste et al. (2023)**	**Miranda et al. (2023)**	**Segur-Ferrer et al. (2024)**	**Stampa et al. (2024)**	**Venkataraman et al. (2024)**	**Ye et al. (2023)**
**Adjekum et al. (2018)**	Bairapareddy, et al. K.C.; Alaparthi, G.K.; Jitendra, R.S.; Prathiksha; Rao, P.P.; Shetty, V.; Chrasekaran, B.We are so close; yet too far: Perceived barriers to smartphone-based telerehabilitation among healthcare providers and patients with Chronic Obstructive Pulmonary Disease in India. Heliyon 2021, 7, e07857. [CrossRef]											■	■	
**Alipour and Hayavi-Haghighi (2021)**	Barney A, Buckelew S, Mesheriakova V, Raymond-Flesch M. The COVID-19 pandemic and rapid implementation of adolescent and young adult telemedicine: challenges and opportunities for innovation. J Adolesc Health 2020; 67 (02) 164-171							■						
**Al-Samarraie et al. (2020)**	Brooks E, Manson SM, Bair B, Dailey N, Shore JH. The diffusion of telehealth in rural American Indian communities: a retrospective survey of key stakeholders. Telemed e-Health. 2012;18(1):60–6.					■	■							
**Babaei et al. (2023)**	Eberly LA, Khatana SAM, Nathan AS. et al. Telemedicine outpatient cardiovascular care during the COVID-19 pandemic: bridging or opening the digital divide?. Circulation 2020; 142 (05) 510-512		■					■						
**Galavi et al. (2022)**	Interian A, King AR, St. Hill LM, Robinson CH, Damschroder LJ. Evaluating the implementation of home-based videoconferencing for providing mental health services. Psychiatr Serv. 2018;69(1):69-75.					■								
**Hui et al. (2022)**	Krenitsky NM, Spiegelman J, Sutton D, Syeda S, Moroz L. Primed for a pandemic: Implementation of telehealth outpatient monitoring for women with mild COVID-19. Semin Perinatol 2020; 44 (07) 151285		■							■				

**Jacob et al. (2020)**	Latifi R, Dasho E, Merrell RC, Lopes M, Azevedo V, Bekteshi F, et al. Cabo Verde telemedicine program: initial results of nationwide implementation. Telemed J E Health 2014 Nov;20(11):1027-1034. [doi: 10.1089/tmj.2014.0026] [Medline: 25083737]					■	■							
**James et al. (2021)**	Li HL, Chan YC, Huang JX, Cheng SW. Pilot study using telemedicine video consultation for vascular patients' care during the COVID-19 period. Ann Vasc Surg 2020; 68: 76-82		■											■
**Kho et al. (2020)**	Lindsay et al (2015) [40], Houston Veterans Affairs, Lindsay JA, Kauth MR, Hudson S, Martin LA, Ramsey DJ, Daily L, et al. Implementation of video telehealth to improve access to evidence-based psychotherapy for posttraumatic stress disorder. Telemed J E Health 2015 Jun;21(6):467-472 [FREE Full text] [doi: 10.1089/tmj.2014.0114] [Medline: 25714664]					■								
**Kowatsch et al. (2019)**	Madden N, Emeruwa UN, Friedman AM. et al. Telehealth uptake into prenatal care and provider attitudes during the COVID-19 pandemic in New York City: a quantitative and qualitative analysis. Am J Perinatol 2020; 37 (10) 1005-1014		■					■						
**Lieneck et al. (2021)**	Martinez RN, Hogan TP, Balbale S, Lones K, Goldstein B, Woo C, et al. Sociotechnical Perspective on Implementing Clinical Video Telehealth for Veterans with Spinal Cord Injuries and Disorders. Telemed J E Health 2017 Jul;23(7):567-576 [FREE Full text] [doi: 10.1089/tmj.2016.0200] [Medline: 28067586]					■	■							
**Mauco et al. (2018)**	Sanders C, Rogers A, Bowen R, Bower P, Hirani S, Cartwright M, et al. Exploring barriers to participation and adoption of telehealth and telecare within the Whole System Demonstrator trial: a qualitative study. BMC Health Services Research. 2012;12:220-. PMID: 22834978. doi: 10.1186/1472-6963-12-220.	■		■										
**Mengiste et al. (2023)**	Shaw RJ, Kaufman MA, Bosworth HB, Weiner BJ, Zullig LL, Lee SY, et al. Organizational factors associated with readiness to implement and translate a primary care based telemedicine behavioral program to improve blood pressure control: the HTN-IMPROVE study. Implement Sci. 2013;8:106.				■		■							
**Miranda et al. (2023)**	Vis C, Bührmann L, Riper H, Ossebaard HC. Health technology assessment frameworks for eHealth: a systematic review. Int J Technol Assess Health Care. Apr 16, 2020;36(3):204-216. [doi: 10.1017/s026646232000015x]								■		■			
**Segur-Ferrer et al. (2024)**	Wood SM, White K, Peebles R. et al. Outcomes of a rapid adolescent telehealth scale-up during the COVID-19 pandemic. J Adolesc Health 2020; 67 (02) 172-178		■					■						
**Stampa et al. (2024)**	Xu H, Huang S, Qiu C. et al. Monitoring and management of home-quarantined Patients With COVID-19 Using a WeChat-based telemedicine system: retrospective cohort study. J Med Internet Res 2020; 22 (07) e19514		■											■

In addition Kowatsch et al. (2019) was included in our study and also in Segur-Ferrer et al. (2024)

References

Adjekum, A., Blasimme, A., & Vayena, E. (2018). Elements of trust in digital health systems: Scoping review. *Journal of Medical Internet Research*, *20*(12), e11254. https://doi.org/10.2196/11254

Alipour, J., & Hayavi-Haghighi, M. H. (2021). Opportunities and Challenges of Telehealth in Disease Management during COVID-19 Pandemic: A Scoping Review. *Applied Clinical Informatics*, *12*(4), 864–876. https://doi.org/10.1055/s-0041-1735181

Al-Samarraie, H., Ghazal, S., Alzahrani, A. I., & Moody, L. (2020). Telemedicine in Middle Eastern countries: Progress, barriers, and policy recommendations. *International Journal of Medical Informatics*, *141*, 104232. https://doi.org/10.1016/j.ijmedinf.2020.104232

Babaei, N., Zamanzadeh, V., Valizadeh, L., Lotfi, M., Samad-Soltani, T., Kousha, A., & Avazeh, M. (2023). A scoping review of virtual care in the health system: infrastructures, barriers, and facilitators. *Home Health Care Services Quarterly*, *42*(2), 69–97. https://doi.org/10.1080/01621424.2023.2166888

Galavi, Z., Montazeri, M., & Ahmadian, L. (2022). Barriers and challenges of using health information technology in home care: A systematic review. *International Journal of Health Planning and Management*, *37*(5), 2542–2568. https://doi.org/10.1002/hpm.3492

Hui, C. Y., Abdulla, A., Ahmed, Z., Goel, H., Habib, G. M. M., Hock, T. T., Khandakr, P., Mahmood, H., Nautiyal, A., Nurmansyah, M., Panwar, S., Patil, R., Rinawan, F. R., Salim, H., Satav, A., Shah, J. N., Shukla, A., Tanim, C. Z. H., Balharry, D., & Pinnock, H. (2022). Mapping national information and communication technology (ICT) infrastructure to the requirements of potential digital health interventions in low and middle-income countries. *Journal of Global Health*, *12*, 04094. https://doi.org/10.7189/jogh.12.04094

Jacob, C., Sanchez-Vazquez, A., & Ivory, C. (2020). Understanding clinicians' adoption of mobile health tools: A qualitative review of the most used frameworks. *JMIR MHealth and UHealth*, *8*(7), e18072. https://doi.org/10.2196/18072

James, H. M., Papoutsi, C., Wherton, J., Greenhalgh, T., & Shaw, S. E. (2021). Spread, Scale-up, and Sustainability of Video Consulting in Health Care: Systematic Review and Synthesis Guided by the NASSS Framework. *Journal of Medical Internet Research*, *23*(1). https://doi.org/10.2196/23775

Kho, J., Gillespie, N., & Martin-Khan, M. (2020). A systematic scoping review of change management practices used for telemedicine service implementations. *BMC Health Services Research*, *20*(1), 815. https://doi.org/10.1186/s12913-020-05657-w

Kowatsch, T., Otto, L., Harperink, S., Cotti, A., & Schlieter, H. (2019). A design and evaluation framework for digital health interventions. *IT - Information Technology*, *61*(5–6), 253–263. https://doi.org/10.1515/ITIT-2019-0019

Lieneck, C., Weaver, E., & Maryon, T. (2021). Outpatient telehealth implementation in the united states during the covid-19 global pandemic: A systematic review. *Medicina (Lithuania)*, *57*(5), 462. https://doi.org/10.3390/medicina57050462

Mauco, K. L., Scott, R. E., & Mars, M. (2018). Critical analysis of e-health readiness assessment frameworks: suitability for application in developing countries. *Journal of Telemedicine and Telecare*, *24*(2), 110–117. https://doi.org/10.1177/1357633X16686548

Mengiste, S. A., Antypas, K., Johannessen, M. R., Klein, J., & Kazemi, G. (2023). eHealth policy framework in Low and Lower Middle-Income Countries; a PRISMA systematic review and analysis. *BMC Health Services Research*, *23*(1), 328. https://doi.org/10.1186/s12913-023-09325-7

iranda, R., Oliveira, M. D., Nicola, P., Baptista, F. M., & Albuquerque, I. (2023). Towards A Framework for Implementing Remote Patient Monitoring From an Integrated Care Perspective: A Scoping Review. *International Journal of Health Policy and Management*, *12*(1), 7299. https://doi.org/10.34172/ijhpm.2023.7299

Segur-Ferrer, J., Moltó-Puigmartí, C., Pastells-Peiró, R., & Vivanco-Hidalgo, R. M. (2024). Methodological Frameworks and Dimensions to Be Considered in Digital Health Technology Assessment: Scoping Review and Thematic Analysis. *Journal of Medical Internet Research*, *26*(1), e48694. https://doi.org/10.2196/48694

Stampa, S., Thienel, C., Tokgöz, P., Razum, O., & Dockweiler, C. (2024). Factors Facilitating and Inhibiting the Implementation of Telerehabilitation—A Scoping Review. *Healthcare (Switzerland)*, *12*(6), 619. https://doi.org/10.3390/healthcare12060619

Venkataraman, A., Fatma, N., Edirippulige, S., & Ramamohan, V. (2024). Facilitators and Barriers for Telemedicine Systems in India from Multiple Stakeholder Perspectives and Settings: A Systematic Review. *Https://Home.Liebertpub.Com/Tmj*, *30*(5), 1341–1356. https://doi.org/10.1089/tmj.2023.0297

Ye, J., He, L., & Beestrum, M. (2023). Implications for implementation and adoption of telehealth in developing countries: a systematic review of China's practices and experiences. *Npj Digital Medicine*, *6*(1), 174. https://doi.org/10.1038/s41746-023-00908-6
