# Supplementary material for: The State of the Art of Telemedicine Implementation Architecture: Rapid Umbrella Review of Systematic Reviews
Source: J Med Internet Res. 2025 Jun 9;27:e70276. doi: 10.2196/70276 (PMC12186003; doi:10.2196/70276)
Supplement: Multimedia Appendix 6 [file jmir_v27i1e70276_app6.pdf]

Multimedia Appendix 6 – Other Characteristics of Included Reviews

Primary Research Characteristics.

Telemedicine Channels & Models	
Telemedicine channels	• telephone calls [1–5].<br>• video-consultations [1–12].<br>• virtual group therapy [13].<br>• email and SMS messaging [1,3,4].<br>• ePrescriptions [6,9].<br>• wearable devices and remote monitoring systems [2–4,7–9,14].<br>• telemedicine for prevention [4,6].<br>• online health management systems and electronic health records [3,7–9,11,13–15].<br>• A generic approach to 'telemedicine' with no channel or models specified [11,13–15].
Popular telemedicine platforms	• Zoom and Zoom for Healthcare, MyChart, Think Research, Novari, FaceTime, Skype, Doxy.me, WeChat. [1,12].
Models	• hub-and-spoke [1,10].<br>• store and forward [2].<br>• patient follow-up [6].
Other digital health interventions	• mHealth [3,5,7–9,14,15].<br>• robotic therapy [7,8].<br>• artificial intelligence solutions [13].<br>• gaming software (e.g., Nintendo Wii©, WiiFit©) [4].<br>• virtual reality technologies [4].<br>• eLearning [3,4].<br>• other forms of personalised medicine [14].
**Clinical Specialities**	
Types of clinical specialities	• primary healthcare (such as weight management, smoking cessation, and management of chronic disease like blood pressure) [1–3,8–12,15,16].<br>• endocrinology including diabetes [1,3,6–10,12,16].<br>• infectious diseases such as COVID-19, vector-borne diseases, and other infectious diseases [1,3,5,6,8,12,15].<br>• maternal and child health [1–3,5,6,8,9,12,15,16].<br>• mental health including psychiatry and neurology [2–6,9,10,12,15,16].<br>• cardiology, heart failure and stoke related diseases [1,3,5–7,9,12,15,16].<br>• cancer treatment [1,3,5–7,10,12].<br>• palliative and aged care [2,3,5,9,10].<br>• emergency and ambulatory care [2,5,6,8,9].<br>• respiratory diseases such as chronic obstructive pulmonary disease [3,7].<br>• internal medicine [2,5,6].<br>• ophthalmology [5,6,12].<br>• orthopaedics [1,4,16].<br>• dermatology [2,5,9,12].<br>• physical medicine and rehabilitation [5,6,10,12].

	• laboratory testing, pathology and cytology [2,5,8].<br>• tele-monitoring after surgery [2,3,5,6].<br>• ear nose and throat diseases (ENT) [1,6].<br>• sexual and reproductive health [9].<br>• urology [6].<br>• substance use recovery [9].<br>• dentistry [5].

Methodological and Theoretical Approaches to the Analysis

Methodology Used for the Systematic Review Evidence Synthesis	
Methodological approach to the review	The evidence synthesis in the SRs comprised qualitative analysis [1–5,7,10–12,14], and mix-method incorporating both qualitative and quantitative analysis [6,8,9,13,15–18].
Analysis process of the systematic review	Many reviews employed an inductive approach to the thematic analysis [1,5,6,11,13,14,16,17]. Others used, a mix of inductive and deductive approaches, deploying a framework synthesis approach, to guide analysis including:<br>• Consolidated Framework for Implementation Research (CFIR) [4,12].<br>• BettereHealth project strategic framework [18].<br>• Nonadoption, Abandonment, and challenges to the Scale-up, Spread, and Sustainability (NASSS) [10].<br>• Prosci 3-Phase Change Process [2].<br>• Sustainable intEgrated chronic care modeLs for multimorbidity: delivery, FInancing, and performancE (SELFIE) framework [3].<br>• Implementation and Operational of Mobile Health project framework and the Extended Technology Acceptance Model of Mobile Telphony [8].<br>• Methodological Guidelines for the study of Materiality and Affordances [9].<br>• Supporting the Use of Research Evidence (SURE) framework [7].<br>• A mix of frameworks used including: Intervention Planning Framework, Intervention Mapping, Medical Research Council Framework and the revised framework, PRECEDE-PROCEED, Multiphase optimisation strategy (MOST), Behaviour Change Wheel, Designing for Behaviour Change, Integrate Design Assess and Share (IDEAS), 6 Steps in Quality Intervention Development [15].
**Methodological Approaches of the Primary Research included in Systematic Reviews**	
Primary research study methodologies	The primary research included in SRs comprised quantitative studies (i.e. randomised control trials and quantitative surveys), qualitative studies (i.e. interviews, focus groups, participatory action research, and case studies), mixed methods studies (i.e. evaluations, assessments and implementation science research) and secondary data analysis (i.e. literature and systematic reviews). In the primary research, there was no evidence of a domination of one methodological approach over another, for instance some SRs comprised more quantitative primary research [5,8], whilst others more qualitative [4,7,13,18], and others included a mix [1,9,10,14].
Primary Research Theoretical underpinnings	Six of the SRs' analysed the theoretical models or frameworks underpinning the primary studies [3,4,9–11,13]. Those that were most frequently present include:<br>• Consolidated Framework for implementation Research [4,9,10].<br>• Reach, Efficacy, Adoption, Implementation, and Maintenance (RE-AIM) framework [2,4].

	• integrated Promoting Action of Research Implementation in Health Services (iPARIHS) framework [4,10].<br>• Technology Acceptance Model [3,9].<br>• Diffusion of Innovation theory [9,10]<br><br>Other theoretical models mentioned:<br>• Unified Theory of Acceptance and Use of Technology [9].<br>• Theoretical Domains Framework [13].<br>• Medical Research Council Framework (MRC) [4].<br>• Sociotechnical theory [9].<br>• Strong Structuration Theory [10].<br>• Organisational Information Technology/Systems Innovation Readiness Scale [11].<br>• Organisational and Functioning Readiness for Change Scale [11].<br>• Digital divide theory [3].<br>• Grol Framework [4].<br>• various other business development theories (such as business to business, and business to consumer models, incorporating activity system theory, and transaction cost theory) [3].

References

1. Babaei N, Zamanzadeh V, Valizadeh L, Lotfi M, Samad-Soltani T, Kousha A, et al. A scoping review of virtual care in the health system: infrastructures, barriers, and facilitators. Home Health Care Serv Q. 2023;42: 69–97. doi:10.1080/01621424.2023.2166888
2. Kho J, Gillespie N, Martin-Khan M. A systematic scoping review of change management practices used for telemedicine service implementations. BMC Health Serv Res. 2020;20: 815. doi:10.1186/s12913-020-05657-w
3. Miranda R, Oliveira MD, Nicola P, Baptista FM, Albuquerque I. Towards A Framework for Implementing Remote Patient Monitoring From an Integrated Care Perspective: A Scoping Review. Int J Health Policy Manag. 2023;12: 7299. doi:10.34172/ijhpm.2023.7299
4. Stampa S, Thienel C, Tokgöz P, Razum O, Dockweiler C. Factors Facilitating and Inhibiting the Implementation of Telerehabilitation—A Scoping Review. Healthcare (Switzerland). 2024;12: 619. doi:10.3390/healthcare12060619
5. Venkataraman A, Fatma N, Edirippulige S, Ramamohan V. Facilitators and Barriers for Telemedicine Systems in India from Multiple Stakeholder Perspectives and Settings: A Systematic Review. https://home.liebertpub.com/tmj. 2024;30: 1341–1356. doi:10.1089/tmj.2023.0297
6. Alipour J, Hayavi-Haghighi MH. Opportunities and Challenges of Telehealth in Disease Management during COVID-19 Pandemic: A Scoping Review. Appl Clin Inform. 2021;12: 864–876. doi:10.1055/s-0041-1735181
7. Galavi Z, Montazeri M, Ahmadian L. Barriers and challenges of using health information technology in home care: A systematic review. International Journal of Health Planning and Management. 2022;37: 2542–2568. doi:10.1002/hpm.3492
8. Hui CY, Abdulla A, Ahmed Z, Goel H, Habib GMM, Hock TT, et al. Mapping national information and communication technology (ICT) infrastructure to

the requirements of potential digital health interventions in low and middle-income countries. J Glob Health. 2022;12: 04094. doi:10.7189/jogh.12.04094
9. Jacob C, Sanchez-Vazquez A, Ivory C. Understanding clinicians' adoption of mobile health tools: A qualitative review of the most used frameworks. JMIR Mhealth Uhealth. 2020;8: e18072. doi:10.2196/18072
10. James HM, Papoutsi C, Wherton J, Greenhalgh T, Shaw SE. Spread, Scale-up, and Sustainability of Video Consulting in Health Care: Systematic Review and Synthesis Guided by the NASSS Framework. J Med Internet Res. 2021;23. doi:10.2196/23775
11. Mauco KL, Scott RE, Mars M. Critical analysis of e-health readiness assessment frameworks: suitability for application in developing countries. J Telemed Telecare. 2018;24: 110–117. doi:10.1177/1357633X16686548
12. Ye J, He L, Beestrum M. Implications for implementation and adoption of telehealth in developing countries: a systematic review of China's practices and experiences. NPJ Digit Med. 2023;6: 174. doi:10.1038/s41746-023-00908-6
13. Segur-Ferrer J, Moltó-Puigmartí C, Pastells-Peiró R, Vivanco-Hidalgo RM. Methodological Frameworks and Dimensions to Be Considered in Digital Health Technology Assessment: Scoping Review and Thematic Analysis. J Med Internet Res. 2024;26: e48694. doi:10.2196/48694
14. Adjekum A, Blasimme A, Vayena E. Elements of trust in digital health systems: Scoping review. J Med Internet Res. 2018;20: e11254. doi:10.2196/11254
15. Kowatsch T, Otto L, Harperink S, Cotti A, Schlieter H. A design and evaluation framework for digital health interventions. IT - Information Technology. 2019;61: 253–263. doi:10.1515/ITIT-2019-0019
16. Lieneck C, Weaver E, Maryon T. Outpatient telehealth implementation in the united states during the covid-19 global pandemic: A systematic review. Medicina (Lithuania). 2021;57: 462. doi:10.3390/medicina57050462
17. Al-Samarraie H, Ghazal S, Alzahrani AI, Moody L. Telemedicine in Middle Eastern countries: Progress, barriers, and policy recommendations. Int J Med Inform. 2020;141: 104232. doi:10.1016/j.ijmedinf.2020.104232
18. Mengiste SA, Antypas K, Johannessen MR, Klein J, Kazemi G. eHealth policy framework in Low and Lower Middle-Income Countries; a PRISMA systematic review and analysis. BMC Health Serv Res. 2023;23: 328. doi:10.1186/s12913-023-09325-7
