# Supplementary material for: The State of the Art of Telemedicine Implementation Architecture: Rapid Umbrella Review of Systematic Reviews
Source: J Med Internet Res. 2025 Jun 9;27:e70276. doi: 10.2196/70276 (PMC12186003; doi:10.2196/70276)
Supplement: Multimedia Appendix 7 [file jmir_v27i1e70276_app7.pdf]

Multimedia Appendix 7 – Summary of Knowledge Tool Outputs of Reviews

Group1 - Implementation Frameworks and Models	
Jacob et al. (2020) [1]	Exploring theoretical frameworks of implementation, this knowledge tools comprises a segmented circular pie chart which consists of three constructs:<br>• organisational and policy,<br>• technical and material,<br>• social and personal,<br>These are further divided into fourteen sub-constructs, some of which are further elaborated into sub-domains.
James et al. 2021) [2]	This review focusing on spread and scale, comprises a layered seven dimension, dynamic and directional tool, comprising implementation constructs of:<br>• condition - health and sociocultural,<br>• technology,<br>• value proposition,<br>• adopter system,<br>• the health organisation,<br>• wider system,<br>• continuous embedding and adaptation over time.<br>Each of the seven constructs is further defined.
Kho et al. (2020) [3]	This review exploring implementation from a change management perspective uses a stepwise approach. The model comprises three dimensions:<br>• Preparing for change<br>• Managing change<br>• Reinforcing change<br>These dimensions are further delineated into (i) change steps, (ii) strategic practices and (iii) operational practices.
Kowatsch et al. (2019) [4]	The findings of this review which focuses on prototyping implementation design, takes a process, stepwise approach, identifying four phases of implementation:<br>• Preparation phase<br>• Optimisation Phase<br>• Evaluation Phase<br>• Implementation Phase<br><br>Each Phase is further defined in terms of (i) goals and tasks, (ii) technical maturity, (iii) evaluation criteria, and (iv) implementation barriers.
Mengiste et al. (2023) [5]	With a focus on implementation policy, this review takes a stepwise-process approach identifying four steps of implementation:<br>• Step 1: Before policy creation<br>• Step 2: Develop policy based on realistic expectations.<br>• Step 3: Develop guidelines, framework, roadmap for implementation.<br>• Step 4: Evaluation and adaptation<br><br>In addition, the review identifies three dimensions of implementation:<br>• Technical: Interoperability, Standardisation, Infrastructure<br>• Human: Capacity building, Empowerment, User friendliness<br>• Policy: Roadmap, Strategic Implementation, Legal and Financial.

Miranda et al. (2023) [6]	In this review examining remote patient monitoring and integrated care, the knowledge tool output is a circular three-tier model consisting of:<br>• Elementary design elements (including patient education and promotion, multidisciplinary workforce, technology, and measurement of health indicators.)<br>• Integrated care delivery elements<br>• Added value elements.
Group 2 - Mapping Implementation Theoretical Models	
Mauco et al. (2018) [7]	Readiness assessment frameworks are synthesised into eight dimensions of readiness:<br>• Organisational readiness<br>• Technological/infrastructural readiness<br>• Healthcare provider readiness<br>• Engagement readiness<br>• Societal readiness<br>• Core readiness<br>• Government readiness<br>• Public/Patient readiness
Segur-Ferrer et al. (2024) [8]	This review which maps different technology assessment models, synthesises the constructs into a hierarchical, three levels diagram:<br>• Level 1 comprising 12 domains,<br>• Level 2 comprising 38 dimensions,<br>• Level 3 comprising 11 subdimensions.<br><br>Level 1 includes the implementation dimensions of: description of the health problem, description of the technology, content, safety, clinical efficacy and effectiveness, economic aspects, human and sociocultural aspects, ethical aspects, legal and regulatory aspects, technical aspects, and post monitoring
Group 3 - Matrix of Implementation Factors	
Adjekum et al. (2018) [9]	The construct of 'trust' is explored in this review, which defines three levels of classification of implementation:<br>• Personal elements:<br>• Technological elements<br>• Institutional elements<br><br>These are further delineated into enablers of trust and impediments across stakeholder groups of (i) patients, (ii) healthcare providers and (iii) health administrators.
Alipour and Hayavi-Haghighi (2021) [10]	Focusing on COVID-19 pandemic, this review examines telemedicine implementation constructs across the domains of:<br>• Opportunities: (i) clinical, (ii) organisational, (iii) technical, and (iv) social.<br>• Challenges: (i) legal, (ii) clinical, (iii) technical, (iv) organisational , (v) socio/financial, and (vi) data quality.<br><br>The constructs are further defined into 'unique concepts' and 'initial themes'.
Galavi et al. (2022) [11]	In this review, implementation is explored from the perspective of home care and synthesised into the constructs of:<br>• Recipients of care<br>• Providers of care

	• Health system constraints<br><br>Across these dimensions, factors affecting implementation are identified as (i) knowledge and skills, (ii) attitudes regarding programmer acceptability, appropriateness and credibility, (iii) motivation to change or adopt new behaviour, (iv) accessibility of care, (iv) financial resources, (v) human resources, (vi) internal communication, (vii) external communication, (viii) facilities, (ix) relationship with norms and standards, (x) short-term thinking, and (xi) legislation or regulations. These dimensions are further articulated into barriers.
Hui et al. (2022) [12]	This review with a strong emphasis on ‘technical infrastructure’ and ‘context’, identifies a checklist of contextual factors to be considered in implementation consisting of:<br>• Policy, legislation and standards<br>• Infrastructure<br>• Interoperability<br>• Workforce<br>• Adoption costs<br>• Digital inequalities<br>• Encouraging adoption features.
Lieneck et al. (2021) [13]	Outpatient telehealth implementation is explored in this review which examines implementation factors using a horizontal list diagram, from the perspective of:<br>• Facilitator themes – including (i) patient engagement, (ii) operational workflow and organisational readiness, and (iii) regulatory changes and reimbursement parity.<br>• Barrier themes – including (i) patient limitations, (ii) lack of clinical care guidelines, and (iii) training, technology, and financial considerations.
Stampa et al. (2024) [14]	This study reviews implementation with a focus on telerehabilitation using the Consolidated Framework for Implementation Research (CFIR) model across the dimensions and constructs of<br>• Innovation: (i) evidence base, (ii) relative advantage, (iii) adaptability, (iv) complexity, (v) design, and (vi) cost<br>• Inner setting: (i) physical infrastructure, (ii) information technology infrastructure, (iii) work infrastructure, (iv) relational connections, (v) communication, (vi) human-equality-centredness, (vii) recipient centredness, (viii) learning centredness, (ix) compatibility, (x) relative priority, (xi) incentive system, (xii) available resources, and (xiii) access to knowledge.<br>• Outer setting: (i) local conditions, (ii) partnership and connections, (iii) policies and laws, (iv) financing, and (v) external pressure.<br>• Individual: (i) high-level leaders, (ii) implementation team members, (iii) other implementation support, (iv) capability, (v) opportunity, and (vi) motivation.<br>• Implementation process: (i) teaming, (ii) planning, (iii) engaging, and (iv) reflecting and evaluation.
Venkataraman et al. (2024) [15]	Implementation from the perspective of stakeholders and setting is explored in this review, which identifies the implementation constructs of:<br>• Infrastructural factors<br>• Sociocultural factors<br>• Regulatory and financial factors.

	Each factor is explored from the perspective of facilitators and barriers and across stakeholder groups of (i) doctors and healthcare providers, (ii) patients and patient caregivers, (iii) society, (iv) healthcare organisations.
Ye et al. (2023) [16]	In this review, implementation constructs are mapped against the Consolidated Framework for Implementation Research (CFIR) comprising:<br>• Outer setting,<br>• Inner setting<br>• Intervention characteristic<br>• Characteristics of individuals<br>• Process<br><br>Across these dimensions barriers are identified along with successful experience and recommendations.
Group 4 - Strategic Recommendations	
Al-Samarraie et al. (2020) [17]	Telemedicine implementation in the Middle East is explored in this review. The knowledge tool output synthesis identifies strategic recommendations addressing the barriers in the dimensions of:<br>• Organisational barriers<br>• Legal barriers<br>• Individual barriers<br>• Financial barriers<br>• Technological barriers<br>• Cultural and societal barriers<br>• Legal and Regulatory barriers
Babaei et al. (2023) [18]	Exploring virtual care, this review identifies implementation strategic recommendations as follows:<br>• Virtual care delivery challenges: including lack of information technology facilities and equipment in the organisation, ethical and social issues, unfamiliarity of healthcare providers and patients with technology, unclear healthcare service type and provision, difficulties of changing working conditions and incomplete two-way interaction between virtual care providers and patients,<br>• Facilitators for virtual care implementation were identified as: develop the Information and Communication Technology infrastructure, organisational support to facilitate the launch of virtual care programs, develop a framework for guidance and further training, and participation of consumers in the virtual care policy and planning.

References

1. Jacob C, Sanchez-Vazquez A, Ivory C. Understanding clinicians' adoption of mobile health tools: A qualitative review of the most used frameworks. JMIR Mhealth Uhealth. 2020;8: e18072. doi:10.2196/18072
2. James HM, Papoutsi C, Wherton J, Greenhalgh T, Shaw SE. Spread, Scale-up, and Sustainability of Video Consulting in Health Care: Systematic Review and Synthesis Guided by the NASSS Framework. J Med Internet Res. 2021;23. doi:10.2196/23775

3. Kho J, Gillespie N, Martin-Khan M. A systematic scoping review of change management practices used for telemedicine service implementations. BMC Health Serv Res. 2020;20: 815. doi:10.1186/s12913-020-05657-w
4. Kowatsch T, Otto L, Harperink S, Cotti A, Schlieter H. A design and evaluation framework for digital health interventions. IT - Information Technology. 2019;61: 253–263. doi:10.1515/ITIT-2019-0019
5. Mengiste SA, Antypas K, Johannessen MR, Klein J, Kazemi G. eHealth policy framework in Low and Lower Middle-Income Countries; a PRISMA systematic review and analysis. BMC Health Serv Res. 2023;23: 328. doi:10.1186/s12913-023-09325-7
6. Miranda R, Oliveira MD, Nicola P, Baptista FM, Albuquerque I. Towards A Framework for Implementing Remote Patient Monitoring From an Integrated Care Perspective: A Scoping Review. Int J Health Policy Manag. 2023;12: 7299. doi:10.34172/ijhpm.2023.7299
7. Mauco KL, Scott RE, Mars M. Critical analysis of e-health readiness assessment frameworks: suitability for application in developing countries. J Telemed Telecare. 2018;24: 110–117. doi:10.1177/1357633X16686548
8. Segur-Ferrer J, Moltó-Puigmartí C, Pastells-Peiró R, Vivanco-Hidalgo RM. Methodological Frameworks and Dimensions to Be Considered in Digital Health Technology Assessment: Scoping Review and Thematic Analysis. J Med Internet Res. 2024;26: e48694. doi:10.2196/48694
9. Adjekum A, Blasimme A, Vayena E. Elements of trust in digital health systems: Scoping review. J Med Internet Res. 2018;20: e11254. doi:10.2196/11254
10. Alipour J, Hayavi-Haghighi MH. Opportunities and Challenges of Telehealth in Disease Management during COVID-19 Pandemic: A Scoping Review. Appl Clin Inform. 2021;12: 864–876. doi:10.1055/s-0041-1735181
11. Galavi Z, Montazeri M, Ahmadian L. Barriers and challenges of using health information technology in home care: A systematic review. International Journal of Health Planning and Management. 2022;37: 2542–2568. doi:10.1002/hpm.3492
12. Hui CY, Abdulla A, Ahmed Z, Goel H, Habib GMM, Hock TT, et al. Mapping national information and communication technology (ICT) infrastructure to the requirements of potential digital health interventions in low and middle-income countries. J Glob Health. 2022;12: 04094. doi:10.7189/jogh.12.04094
13. Lieneck C, Weaver E, Maryon T. Outpatient telehealth implementation in the united states during the covid-19 global pandemic: A systematic review. Medicina (Lithuania). 2021;57: 462. doi:10.3390/medicina57050462
14. Stampa S, Thienel C, Tokgöz P, Razum O, Dockweiler C. Factors Facilitating and Inhibiting the Implementation of Telerehabilitation—A Scoping Review. Healthcare (Switzerland). 2024;12: 619. doi:10.3390/healthcare12060619
15. Venkataraman A, Fatma N, Edirippulige S, Ramamohan V. Facilitators and Barriers for Telemedicine Systems in India from Multiple Stakeholder Perspectives and Settings: A Systematic Review. https://home.liebertpub.com/tmj. 2024;30: 1341–1356. doi:10.1089/tmj.2023.0297
16. Ye J, He L, Beestrum M. Implications for implementation and adoption of telehealth in developing countries: a systematic review of China's practices

and experiences. NPJ Digit Med. 2023;6: 174. doi:10.1038/s41746-023-00908-6

17. Al-Samarraie H, Ghazal S, Alzahrani AI, Moody L. Telemedicine in Middle Eastern countries: Progress, barriers, and policy recommendations. Int J Med Inform. 2020;141: 104232. doi:10.1016/j.ijmedinf.2020.104232
18. Babaei N, Zamanzadeh V, Valizadeh L, Lotfi M, Samad-Soltani T, Kousha A, et al. A scoping review of virtual care in the health system: infrastructures, barriers, and facilitators. Home Health Care Serv Q. 2023;42: 69–97. doi:10.1080/01621424.2023.2166888
